# Association of maize (*Zea mays* L.) senescence with water and nitrogen utilization under different drip irrigation systems

**DOI:** 10.3389/fpls.2023.1133206

**Published:** 2023-03-17

**Authors:** Yang Wu, Fanyun Yao, Yongjun Wang, Lin Ma, Xiangnan Li

**Affiliations:** ^1^ Institute of Jiangxi Oil-tea Camellia, Jiujiang University, Jiujiang, China; ^2^ Institute of Agricultural Resource and Environment, Jilin Academy of Agricultural Sciences, Changchun, China; ^3^ Institute of Animal Science, Chinese Academy of Agricultural Sciences, Beijing, China; ^4^ Northeast Institute of Geography and Agroecology, Chinese Academy of Sciences, Changchun, China; ^5^ College of Advanced Agricultural Sciences, University of Chinese Academy of Sciences, Beijing, China

**Keywords:** leaf nitrogen, live root, green leaf area, water use efficiency, nitrogen use efficiency, drip irrigation

## Abstract

**Introduction:**

Drip irrigation is an efficient water-saving system used to improve crop production worldwide. However, we still lack a comprehensive understanding of maize plant senescence and its association with yield, soil water, and nitrogen (N) utilization under this system.

**Methods:**

A 3-year field experiment in the northeast plains of China was used to assess four drip irrigation systems: (1) drip irrigation under plastic film mulch (PI); (2) drip irrigation under biodegradable film mulch (BI); (3) drip irrigation incorporating straw returning (SI); and (4) drip irrigation with the tape buried at a shallow soil depth (OI), and furrow irrigation (FI) was used as the control. The plant senescence characteristic based on the dynamic process of green leaf area (GLA) and live root length density (LRLD) during the reproductive stage, and its correlation with leaf N components, water use efficiency (WUE), and N use efficiency (NUE) was investigated.

**Results:**

PI followed by BI achieved the highest integral GLA and LRLD, grain filling rate, and leaf and root senescence rate after silking. Greater yield, WUE, and NUE were positively associated with higher N translocation efficiency of leaf protein responding for photosynthesis, respiration, and structure under PI and BI; whereas, no significant differences were found in yield, WUE, and NUE between PI and BI. SI effectively promoted LRLD in the deeper 20- to 100-cm soil layers, prolonged the GLA and LRLD persistent durations, and reduced the leaf and root senescence rates. The remobilization of non-protein storage N was stimulated by SI, FI, and OI, which made up for the relative inadequacy of leaf N.

**Discussion:**

Instead of persistent GLA and LRLD durations and high translocation efficiency of non-protein storage N, fast and large protein N translocation from leaves to grains under PI and BI was found to facilitate maize yield, WUE, and NUE in the sole cropping semi-arid region, and BI was recommend considering that it can reduce plastic pollution.

## Introduction

Maize (*Zea mays* L.) is a major cereal crop that widely cultivated globally for grain, forage, and industrial raw material. The northeast plain of China (NEP) accounts for 34% maize production in the country. However, extreme weather events are increasing along with global climate changes, and drought disasters have been a growing threat to maize production. It is therefore imperative to implement water-saving agricultural practices (Lobell et al., 2014). Drip irrigation is one of the most water-efficient irrigation strategies, capable of prominently increasing crop yields and alleviating soil salinization in areas affected by drought. Meanwhile, drip irrigation coupled with plastic film mulching (PI) has become popular strategies used in NEP maize production since 2012 to help manage the impacts of increasing droughts by inhibiting excess evaporation (Zhang et al., 2018). Numerous studies have demonstrated that PI is effective in promoting crop growth, water use efficiency (WUE), and nitrogen use efficiency (NUE) by improving soil hydrothermal conditions, especially in arid or semi-arid regions with low annual average temperatures (Qin et al., 2016; Xue et al., 2017; Wang et al., 2021; Zhang et al., 2022). More recently, drip irrigation combined with biodegradable film mulching (BI) or straw returning to field (SI) as wells as drip irrigation with the tape buried to a shallow soil depth (OI) are methods employed to prevent plastic film pollution and achieve both economic and environmental benefits.

For annual crops, senescence is the last phase of the plant life cycle, comprising the reproductive phase and post-fertilization grain filling. Over 80 years ago, it was discovered that most of the diversity in crop yield is a consequence of variation in the leaf area duration rather than the photosynthesis rate (Heath and Gregory, 1938). Root senescence is strongly linked with leaf senescence *via* nutrient translocation and hormone signaling (Glanz-Idan et al., 2020; Zhu et al., 2021). Previous studies mostly focused on leaf senescence and indicated that stay-green genotypes with lagging senescence exhibit higher nitrogen (N) uptake and grain production than non–stay-green genotypes, especially under drought or low N stresses (Borrell et al., 2000; Gregersen et al., 2013; Kamal et al., 2018; Liu et al, 2021). Except for genetic background, plant senescence is also affected by agronomic management In addition to genetic background, plant senescence is also affected by agronomic management. Appropriate irrigation scheduling will prolong the leaf photosynthetic duration and increase crop yields by alleviating drought stresses (Si et al., 2023). Acciaresi et al. (2014) found that delayed leaf senescence at lower canopy levels was not associated with an increase in post-silking carbon (C) accumulation and may therefore be unproductive for maize under non-stressing conditions. In contrast, Li et al. (2022) found that high-density maize planting was associated with increased N uptake, C assimilation, root function, and yield, owing to the delayed post-silking senescence in lower leaves. Furthermore, plant senescence is directly associated with large N translocation from leaf to grains. Leaf N allocation has great importance in the photosynthetic capacity and the balance of N and C (Liu et al., 2018a; Evans and Clarke, 2019; Mu and Chen, 2021), and it can be classified in detail by function as photosynthetic N, structural N, respiration N, and storage N (Xu et al., 2012; Ali et al., 2016). However, the response of leaf N components to plant senescence process is still unclear.

Film mulching, or film mulching combined with irrigation, can greatly facilitate N utilization (Gu et al., 2021; Liao et al., 2022). Some research studies have suggested that high soil temperature under PI-limited root activity decreases N supply to the canopy and thus accelerates plant senescence (Yang et al., 2016). However, we still lack a comprehensive understanding of leaf and root senescence patterns under varied water-saving irrigation systems as well as the impact of plant senescence on yield, soil water, and N utilization. To address these knowledge gaps, we conducted a 3-year field experiment in NEP consisting of four water-saving drip irrigation treatments (PI, BI, SI, and OI) applied to maize planting. The purpose of this study was to identify the plant senescence characteristic based on the dynamic process of green leaf area (GLA) and live root length density (LRLD) during the reproductive stage, as well as its association with leaf N components, grain filling, yield, WUE, and NUE. The study contributes vital information to improve evaluation of maize productivity under different drip irrigation systems in a semi-arid region.

## Material and methods

### Site description

The experiment was conducted during 2016–2018 at the Taonan farm research station (45° 20′N, 122° 47′E), Jilin Province, China. In the 0- to 100-cm soil layer, the soil was clay loam with a mean bulk density of 1.5 g cm^–3^, a field capacity of 22.7% (weight %), and a wilting coefficient of 11.8% (weight %). The organic matter content and the available N, P, and K were 8.8, 50.4, 20.0, and 90.5 mg kg^–1^, respectively, which was determined according to Arif et al. (2017). Over the last 35 years, the annual mean sunshine duration was 2,817.2 h, the annual mean pan evaporation was 1,928 mm, the frost-free season was 140 days, the annual mean temperature was 6.0°C, and the annual mean precipitation was 419.7 mm. The precipitation and air temperature distributions during the experimental period are shown in **Supplementary Figure 1** and **Supplementary Table 1**.

### Experimental design

Four treatments were set as follows: (1) drip irrigation under plastic film mulch (PI), (2) drip irrigation under biodegradable film mulch (BI), (3) drip irrigation incorporating straw returning (SI), and (4) drip irrigation with the tape buried at a shallow soil depth (OI). In addition, traditional furrow irrigation (FI) was used as the control. The experiment employed a completely randomized design with three replicates, and each plot area measured 255 m^2^ (8.5 × 30 m). Plastic film (polyethylene clear film, 0.9 m wide × 0.008 mm thick; produced by Jilin Difu Agricultural Technology Co. Ltd., Jilin, China) and biodegradable film (polylactic clear film, 0.9 m wide × 0.008 mm thick; produced by Jilin Difu Agricultural Technology Co. Ltd., Jilin, China) were used to cover the surface of the planting ridges. Varying levels of damage to the biodegradable film were observed in August, and the film was completely degraded after crop harvest. The planting schematic diagram for the different treatments is shown in **Supplementary Figure 2**. Maize cultivar “Fumin 985” (dent type) was sown at a rate of 77,000 plants ha^−1^ based on the local planting density.

Under SI treatment, maize straw produced in the identical plot areas (9,000 kg ha^−1^) was cut to lengths of ~10 cm and scattered over the ground evenly. Then, the straw was returned into a 20-cm deep soil layer by using a 110-horsepower tractor after harvest (ca. October 1~10). Except for SI, the straw under other treatments was completely removed from the previous season. In the OI and SI treatments, drip tape was buried at a soil depth of 5–10 cm to prevent evaporation of soil water. Film mulching, drip tape laying, fertilizer application, and seed sowing were performed synchronously by using a multi-functional machine equipped with a 60-horsepower tractor. The drip tape was taken away after harvest by using a recovery machine equipped with a 15-horsepower tractor. All the machines were provided by Jilin Province Kangda Agricultural Machinery Co. Ltd. in Jilin, China.

Fertilizer consisted of N (210 kg ha^−1^), phosphorus pentoxide (105 kg ha^−1^), and potassium oxide (90 kg ha^−1^) applied one time before sowing for each treatment. Supplemental irrigation at critical water demand periods was considered the most efficient way to meet the soil water deficit and is a method that can be easily adopted by local farmers (Gao et al., 2017; Yang et al., 2022). The soil was very dry before sowing in the experimental region (about 50% of the field capacity in the 0- to 20-cm soil layer). To guarantee seed germination and seedling growth, 55 mm of irrigation water was applied on 02 May, 30 April, and 04 May, in the 2016, 2017, and 2018 planting seasons, respectively. Another 40, 30, and 20 mm of irrigation water was applied for each treatment at the jointing, tasseling, and filling stages, respectively. The irrigation amount was determined as the difference between crop water demand (*ET*_C_) and effective precipitation during the past 6 years (Wu et al., 2019). *ET*_C_ determination was based on the Food and Agriculture Organization (FAO) Penman–Monteith equation (Allen et al., 1998). The effective precipitation was the fraction of the precipitation excluding surface runoff, deep percolation, or evaporation, and it was calculated by using the method of Döll and Siebert (2002). The specific irrigation time and precipitation amount between each irrigation event is described in **Supplementary Table 2**. The drip irrigation tape was placed in the middle of planting rows, and the spacing distance was 130 cm (**Supplementary Figure 2**). The tape was 16 mm in diameter with an emitter spacing of 30 cm, and the flow rate of the emitter was 3 L h^−1^ at a working pressure of 0.1 MPa. The irrigation rate was recorded using a precise water meter. The maize seeds were sown on 04 May, 01 May, and 06 May in the 2016, 2017, and 2018, respectively. Growth and developmental progress for each treatment are listed in **Table 1**.

**Table 1:** Developmental progress with different drip irrigation treatments (date, days after sowing).

Stage	Treatments ^a^	2016	2017	2018
Silking	PI	7/17 (74)	7/13 (73)	7/18 (73)
	BI	7/21 (78)	7/18 (78)	7/22 (77)
	SI/OI/FI	7/26 (83)	7/25 (85)	7/28 (83)
Maturity	PI	9/20 (139)	9/16 (138)	9/18 (135)
	BI	9/23 (142)	9/19 (141)	9/20 (137)
	SI/OI/FI	9/27 (146)	9/24 (146)	9/27 (144)

a PI and BI represent drip irrigation under plastic film mulch and biodegradable film mulch, respectively; SI, drip irrigation incorporating straw returning; OI, drip irrigation with the tape buried at a shallow soil depth; FI, furrow irrigation.

### Grain weight, yield, and NUE

Fifty ears that silked on the same day with uniform growth were tagged for each plot. Three tagged ears from each plot were sampled at 10-day intervals from the beginning of the first treatment entered the silking stage. The total number of grains was determined, and the grains were oven-dried at 65°C until constant weight. The 100-kernel weight was calculated until maturity for each treatment. At harvest, four representative, undamaged lines were selected from each plot, and 15 random plants in each line were harvested. The numbers of seeds per ear and the seed weight (14% standard water content) were determined to estimate the yield.

Three maize plants in each plot were sampled to measure biomass and N uptake at silking and maturity stages. Thereinto, roots were sampled every 10 cm in the 0- to 40-cm soil layer, and every 20 cm in the 40- to 100-cm soil layer. The root sampling position was determined on the basis of root distribution area: 65 × 20 × 100 cm (**Supplementary Figure 2**), using a 100/50-mm-diameter steel core-sampling drill. Root samples were carefully washed, and any non-root impurities were carefully removed. Plant samples (roots and aboveground parts) were then oven-dried to constant weights at 65°C to calculate the dry weights. After weighing, the dry samples were ground and passed through a 1-mm sieve, and the N concentration was measured using the micro-Kjeldahl method (CN61M/KDY-9820; Beijing, China) (Li et al., 2017a). Plant N uptake was calculated as the product of plant N concentration and dry matter weight. N translocation amount was calculated as the difference of N uptake between silking and maturity. In addition, N translocation efficiency was defined as the N translocation amount divided by N uptake at silking. The NUE was calculated as the ratio of yield relative to N uptake amount of the whole plant at maturity (Fu et al., 2017).

### Soil water and WUE

Soil was sampled to a depth of 100 cm, following previous studies conducted in the similar semi-arid irrigation regions (Li et al., 2017b; Wang et al., 2019). Soil was sampled every 10 cm in the 0- to 40-cm soil layer, and every 20 cm in the 40- to 100-cm soil layer. According to the root distribution area, soil was sampled at three positions (**Supplementary Figure 2**). The average value of the three horizontal position samples was used to analyze the soil water profile. Soil water was measured by drying the soil to a constant weight at 105°C and then weighing. The field evapotranspiration (*ET*) value was calculated using the soil water balance equation described in Wu et al. (2021). Briefly, *ET* (mm) was equivalent to the sum of precipitation, irrigation, and the difference in soil water storage during a certain growth period. WUE was calculated as the ratio of the grain yield relative to ET during the entire growth period (Payero et al., 2008).

### Live root length density and green leaf area

Fifteen representative plants per plot were tagged to measure the total leaf area every 10 days, starting when the first treatment entered into silking. Individual leaf area was calculated as the product of leaf length and width multiplied by 0.75. Subsequently, GLA was estimated visually until the canopy of all the plants fully turned yellow (Lisanti et al., 2013). Three maize plants per plot were sampled to measure the dynamics of LRLD every 20 days, from the beginning of the first treatment that entered into silking until the last treatment reached maturity. The same root sampling method was used as previously described (**Supplementary Figure 2**). Functional live roots can be distinguished by staining red using 2,3,5-triphenyltetrazolium chloride (TTC). The detail procedure referred to Stūrīte et al. (2005) is as follows: first, fresh roots were quickly incubated in breakers containing 0.6% (w/v) TTC, 0.06 M phosphate buffer, and 0.05% (v/v) Tween 20, at 24°C for 20 h; then, the roots were scanned with an Epson Perfection scanner, and the live root lengths were analyzed with Win RHIZO (Regent Instruments, Inc., Canada) pixel color classification method. LRLD was calculated by dividing the live root length by the sampling core volume for each of the soil layers.

### Leaf N components

Leaf N components were measured at 20-day intervals once the first treatment entered the silking stage. On the basis of Ali et al. (2016), leaf N components were divided by function as photosynthetic N (*N*_pn_), respiration N (*N*_resp_), structural N (*N*_stru_), and storage N (*N*_store_). Maize is a C4 plant, and thus, we divided *N*_pn_ into five parts: ribulose-1,5-bisphosphate carboxylase (Rubisco) N, phosphoenolpyruvate carboxylase (PEPC) N, pyruvate orthophosphate dikinase (PPDK) N, electron transport/bioenergetics N (*N*_et_, proteins involved in electron transport and light phosphorylated), and light harvesting N (*N*_lh_, proteins for light capture in PSI, PSII, and other light-harvesting pigment protein complexes).

To extract water soluble proteins (*N*_w_) and sodium dodecyl sulfate soluble proteins (*N*_SDS_), frozen leaves were homogenized in extraction buffer and centrifuged following the method of Takashima et al. (2004). Rubisco, PEPC, and PPDK contents were separated using SDS-polyacrylamide gel electrophoresis, resulting in 52 and 15 kDa for Rubisco (Makino et al., 2003), 99 kDa for PEPC (Uedan and Sugiyama, 1976), and 94 kDa for PPDK (Sugiyama, 1973). Coomassie Brilliant Blue R-250–stained bands were washed off with formamide and then detected spectrophotometrically. N content in Rubisco (*N*_Rubisco_), PEPC (*N*_PECP_), and PPDK (*N*_PPDK_) was estimated assuming 16% N in proteins. The sodium dodecyl sulfate insoluble protein N used to build cell walls was identified as structural N (*N*_stru_) according to Takashima et al. (2004). Total leaf N content (*N*_T_) and N content in *N*_SDS_ and *N*_stru_ fractions were also measured using the micro-Kjeldahl method.

*N*_et_, *N*_rep_, and *N*_lh_ were proportional to the maximum electron transport, total respiration rate, and chlorophyll concentration, respectively, and the specific calculation that we used has been described by Liu et al. (2018a). The maximum electron transport rate and total respiration rate (photorespiration rate can be ignored for maize) were measured using the *A*_n_-*C*_i_ curve fitting calculation, according to the mechanistic model developed by Ye et al. (2013). Chlorophyll was extracted using a mixed reagent of acetone and ethyl alcohol in a ratio of 1:1. The concentrations of chlorophyll a and chlorophyll b were measured at 663 and 645 nm, respectively, using a spectrophotometer (Perkinelmer, UK) and were calculated according Arnon (1949).

Apart from *N*_pn_, *N*_resp_, and *N*_stru_, the remaining N can be considered as *N*_store_. Moreover, *N*_store_ included water-soluble protein storage N (*N*_ow_), SDS-soluble protein storage N (*N*_os_), and non-protein storage N (*N*_nop_), where *N*_ow_ was calculated as *N*_w_ minus *N*_Rubisco_, *N*_PECP_, *N*_PPDK_, and *N*_rep_; *N*_os_ was calculated as *N*_SDS_ minus *N*_et_ and *N*_lh_; and *N*_nop_ was calculated as *N*_T_ minus *N*_w_, *N*_SDS_, and *N*_stru_ (Liu et al., 2018a).

### Estimation of senescence and filling traits

Leaf and root senescence dynamics were estimated from a differential logistic function (Equation 1) (van Oosterom et al., 1996) fitted to total plant GLA (*GLA_T_*) and total LRLD in the sampling zone (*LRLD_T_*) per plant, respectively.


Eq. 1 $$y=ae^{b-ct}/\left(1+e^{b-ct}\right)$$

where *y* is *GLA_T_* or *LRLD_T_*; *t* is the number of days after silking; and *a*, *b*, and *c* are constants (*a* is the maximum *y-*value in potential, *b* is related to the onset and terminal of senescence, and *c* is related to senescence rate and duration).

The equation of the Richards function (Equation 2) fitted to the 100-kernel weight was adopted to describe filling dynamics.


Eq. 2 $$y=a/{\left(1+be^{-ct}\right)}^{1/d}$$

where *y* is 100-kernel weight; *t* is the number of days after silking; and *a*, *b*, *c*, and *d* are constants (*a* is the maximum *y-*value in potential, and *b*, *c*, and *d* codetermine the onset, terminal, and rate of filling, respectively).

The specific senescence and filling traits are described in **Table 2**.

**Table 2:** Abbreviations and descriptions of senescence and filling traits estimated from Equations 3 and 4, respectively.

Abbreviations	Traits	Description
*GLA_T_*	Total green leaf area	Green leaf area of total plant
*LRLD_T_*	Total live root length density	Live root length density in total growth zone (65 × 20 × 100 cm)
*GLA_max_*	The maximum *GLA_T_*	The maximum green leaf area of total plant
*LRLD_max_*	The maximum *LRLD_T_*	The maximum live root length density in total growth zone (65 × 20 × 100 cm)
*LT_o_*	Onset of leaf senescence	Time at 95% of the maximum *GLA_T_* in potential
*RT_o_*	Onset of root senescence	Time at 95% of the maximum *LRLD_T_* in potential
*GT_o_*	Onset of active grain filling period	Time at 5% of the maximum 100-kernel weight in potential
*LT_e_*	Terminal of leaf senescence	Time at 1% of the maximum *GLA_T_* in potential
*RT_e_*	Terminal of root senescence	Time at 1% of the maximum *LRLD_T_* in potential
*GT_e_*	Terminal of active grain filling period	Time at 95% of the maximum 100-kernel weight in potential
*D_leaf_*	Green leaf duration	Period from onset to terminal of leaf senescence
*D_root_*	Live root duration	Period from onset to terminal of root senescence
*D_filling_*	Active grain filling duration	Period from onset to terminal of grain filling
*LV_max_*	Maximum leaf senescence rate	Maximum descent rate of *GLA_T_*
*RV_max_*	Maximum root senescence rate	Maximum descent rate of *LRLD_T_*
*GV_max_*	Maximum grain filling rate	Maximum increase rate of 100-kernel weight
*LV_a_*	Average leaf senescence rate	Average descent rate of *GLA_T_* during *D_leaf_*
*RV_a_*	Average root senescence rate	Average descent rate of *LRLD_T_* during *D_root_*
*GV_a_*	Average grain filling rate	Average increase rate of 100-kernel weight during *D_filling_*
*I_leaf_*	Green leaf integral	Cumulative *GLA_T_* from silking to maturity
*I_root_*	Live root integral	Cumulative *LRLD_T_* from silking to maturity
*GWA*	Grain weight increment during active filling period	Accumulation of 100-kernel weight at *D_filling_*

### Statistical analyses

One-way analysis of variance was performed using a general linear model (GLM) of SPSS 19.0 (SPSS, Inc., Chicago, IL, USA). The data from each sampling event for all irrigation treatments were tested using the Duncan’s multiple range tests and different letters at a 0.05 level of probability. Non-linear regression model for the estimation of senescence and filling traits as well as Pearson correlation coefficients were also calculated using SPSS.

## Results

### Yield, WUE, and NUE

**Figure 1:**
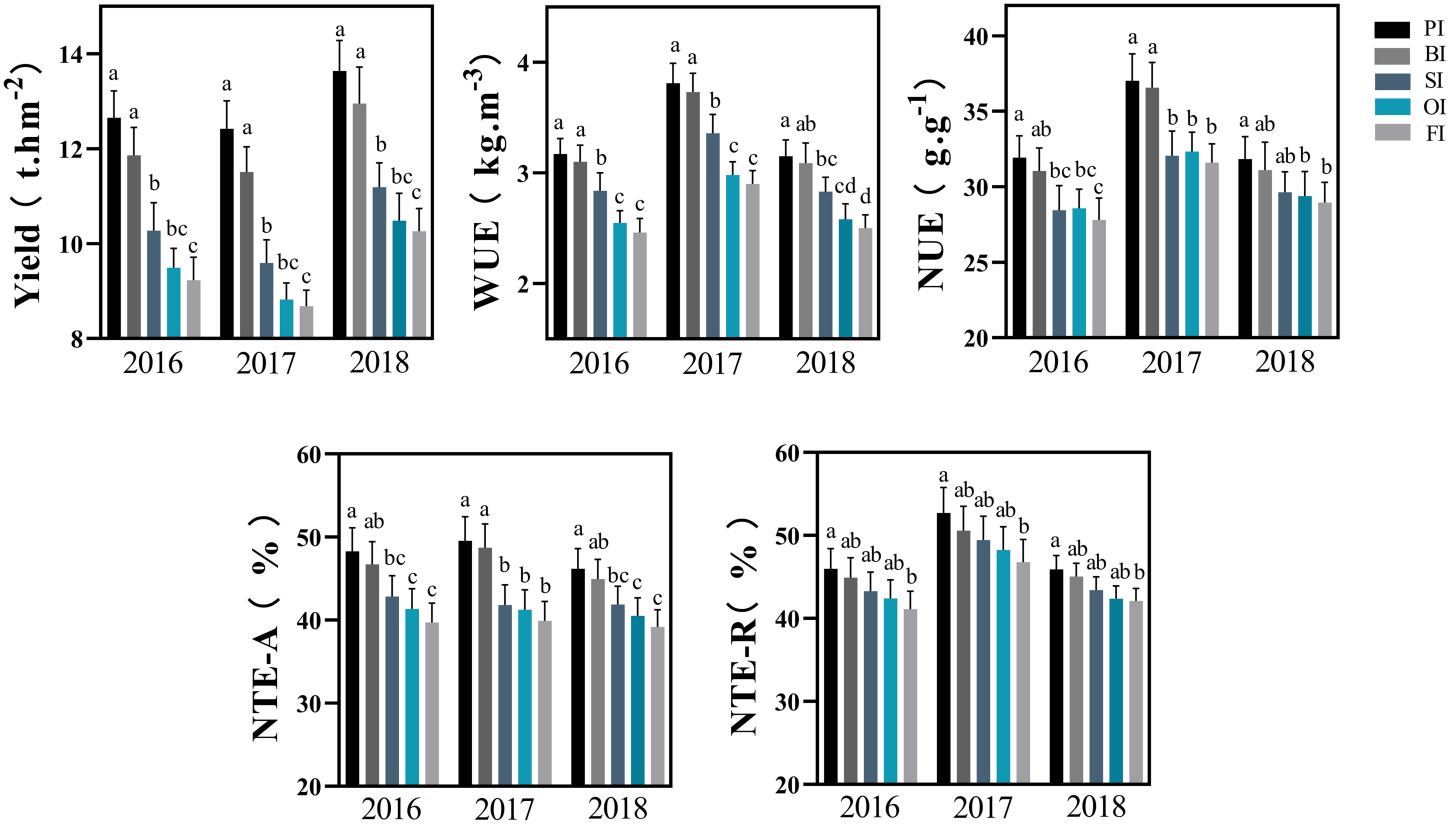
Yield, water, and N use efficiency with the different irrigation treatments. PI and BI represent drip irrigation under plastic film mulch and biodegradable film mulch, respectively; SI, drip irrigation incorporating straw returning; OI, drip irrigation with the tape buried at a shallow soil depth; FI, furrow irrigation. WUE, soil water use efficiency; NTE-A, N translocation efficiency for above ground parts; NTE-R, N translocation efficiency for roots; NUE, N use efficiency. The error bars represent standard deviations. Different lowercase letters indicate significant differences at *P* < 0.05. The data in this figure are presented in **Table S2** for further interpretation.

Film mulching followed by straw returning significantly improved maize yield and WUE under drip irrigation system (**Figure 1**; **Supplementary Table 3**). PI, BI, SI, and OI increased the average yield by 37.48%, 28.93%, 10.27%, and 2.23%, respectively, and increased the average WUE by 28.77%, 26.17%, 14.82%, and 3.08%, respectively, compared with and FI. PI and BI favored N translocation efficiencies for aboveground plant part as well as root and effectively improved NUE compared with other treatments. Whereas, no significant differences were found among SI, OI, and FI for N translocation efficiencies and NUE in different years (*P* > 0.05).

### LRLD distribution

**Figure 2:**
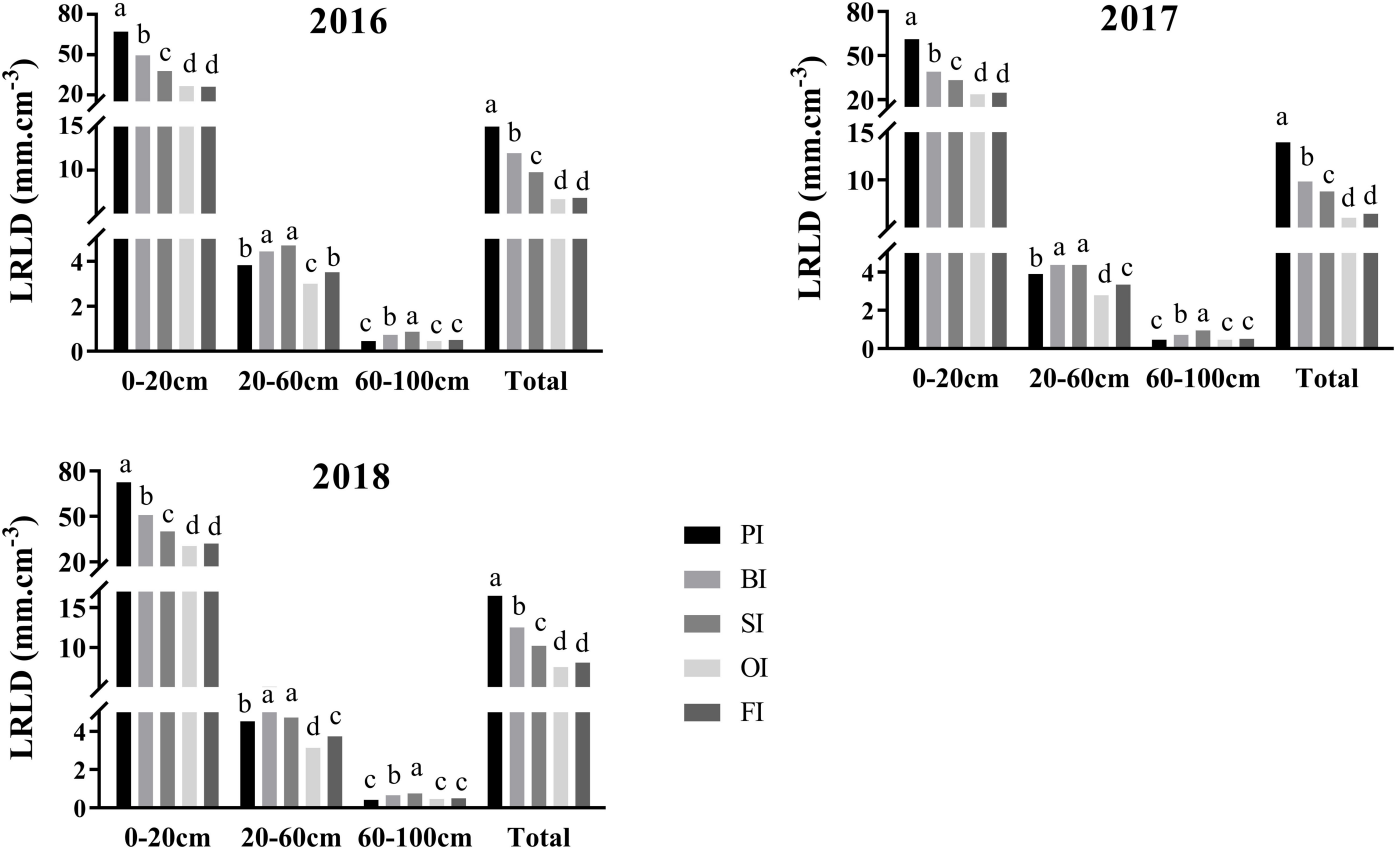
Live root length density (LRLD) distribution at silking in different soil layers. PI and BI represent drip irrigation under plastic film mulch and biodegradable film mulch, respectively; SI, drip irrigation incorporating straw returning; OI, drip irrigation with the tape buried at a shallow soil depth; FI, furrow irrigation. Different lowercase letters indicate significant differences at *P* < 0.05.

PI improved *LRLD*_T_ at silking contributed to the large LRLD in the 0- to 20-cm soil layers (**Figure 2**). SI followed by BI effectively improved LRLD in the deeper 20- to 60-cm and 60- to 100-cm soil layers. There were no significant differences between FI and OI in LRLD in the 0- to 20-cm and 60- to 100-cm soil layers. However, FI significantly increased LRLD in the 20- to 60-cm soil layers in contrast to OI (*P<* 0.05).

### Leaf N components and their translocation efficiencies

**Figure 3:**
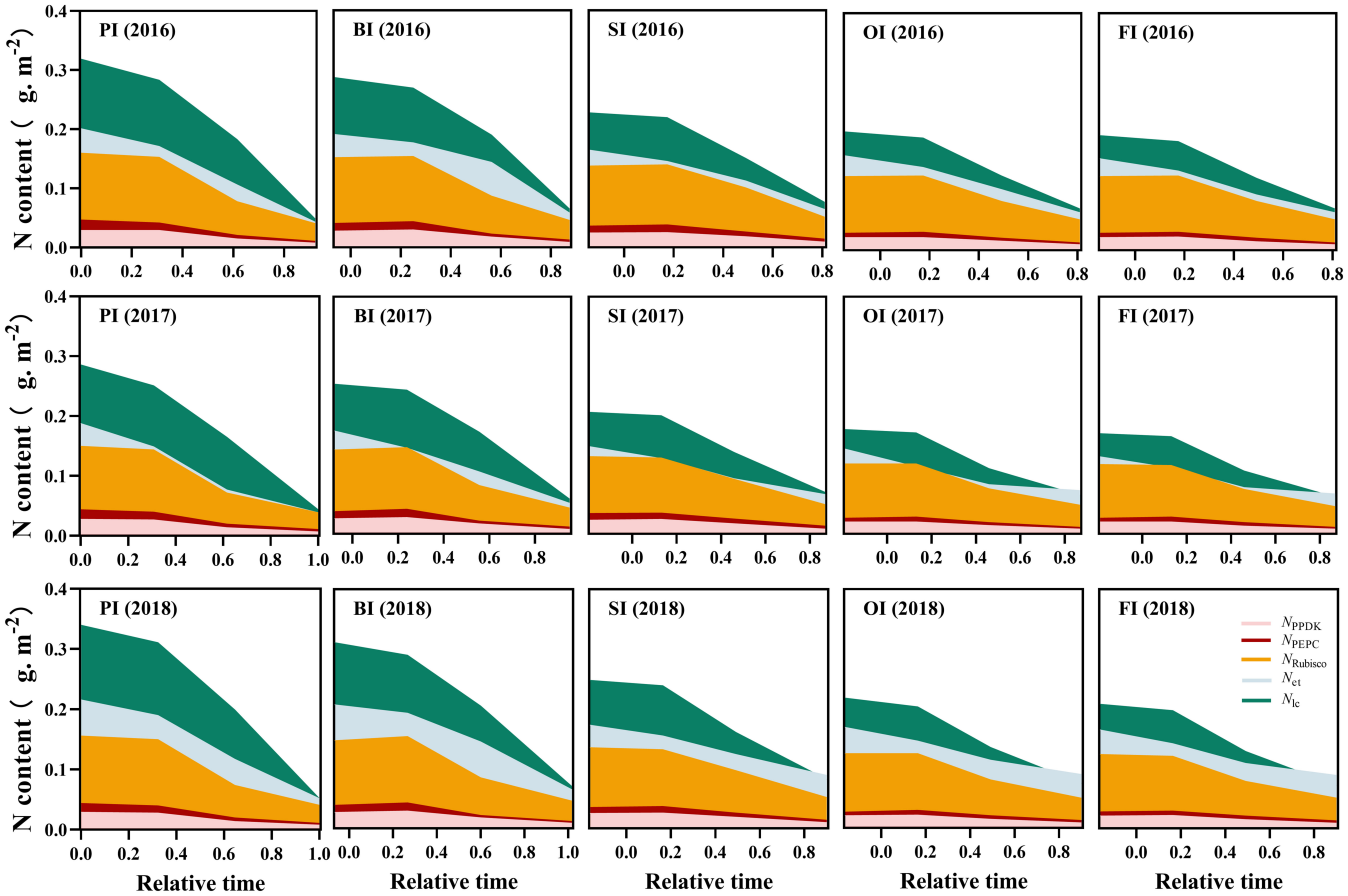
Leaf photosynthetic N dynamics during the reproductive period. PI and BI represent drip irrigation under plastic film mulch and biodegradable film mulch, respectively; SI, drip irrigation incorporating straw returning; OI, drip irrigation with the tape buried to a shallow soil depth; FI, furrow irrigation. Relative time is the ratio of days after silking to the duration from silking to maturity. *N*_PPDK_, *N*_PECP_, and *N*_Rubisco_ represent N content in PPDK, PEPC, and Rubisco protein, respectively. *N*_lh_, protein N responsible for light harvesting system. *N*_et_, protein N responsible for electron transport/ bioenergetics. The data in this figure are presented in **Table S4** for further interpretation.

**Figure 4:**
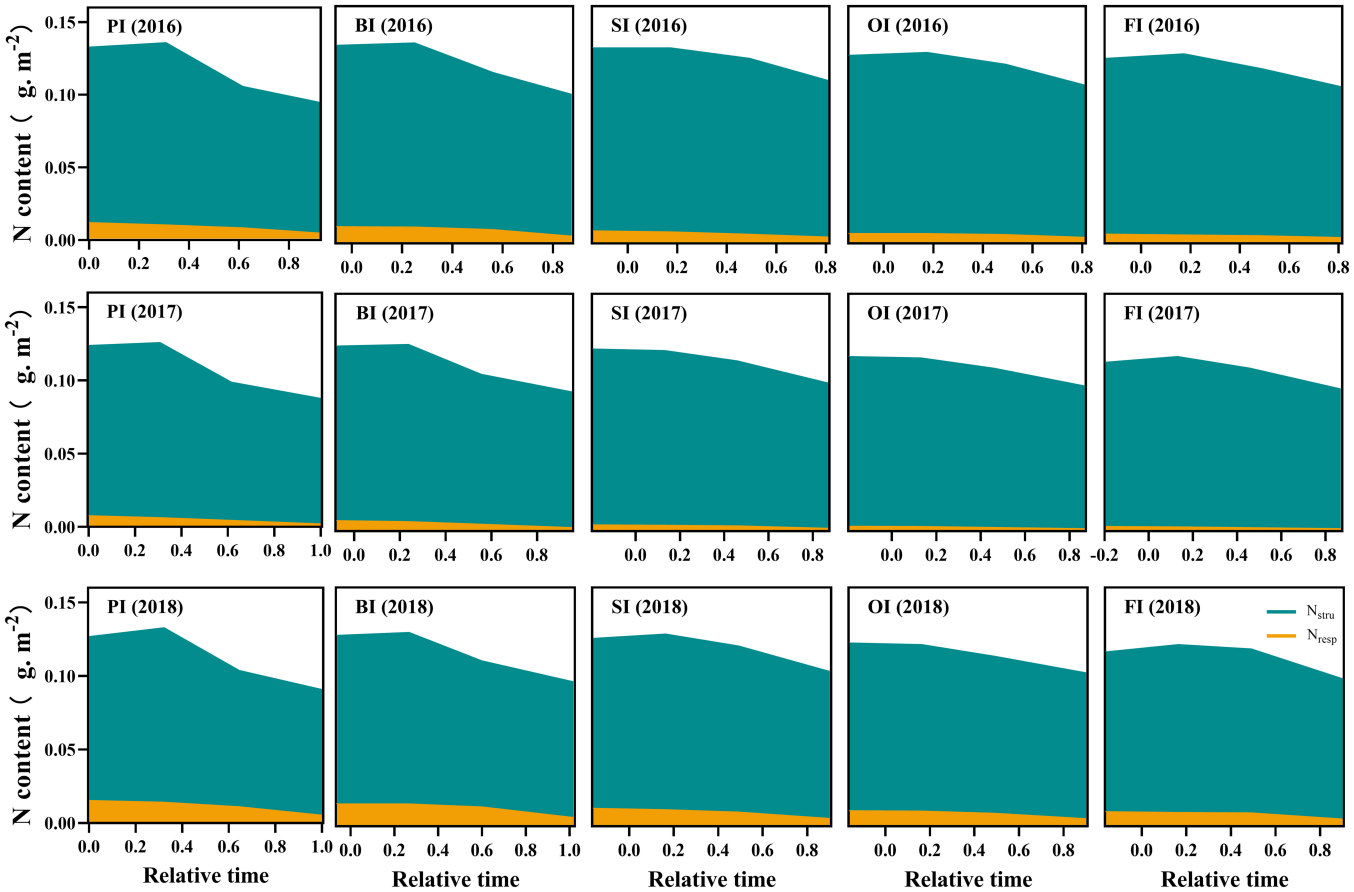
Leaf respiration protein N (*N*_resp_) and structural protein N (*N*_stru_) dynamics during the reproductive period. PI and BI represent drip irrigation under plastic film mulch and biodegradable film mulch, respectively; SI, drip irrigation incorporating straw returning; OI, drip irrigation with the tape buried to a shallow soil depth; FI, furrow irrigation. Relative time is the ratio of days after silking to the duration from silking to maturity. The data in this figure are presented in **Table S4** for further interpretation.

**Figure 5:**
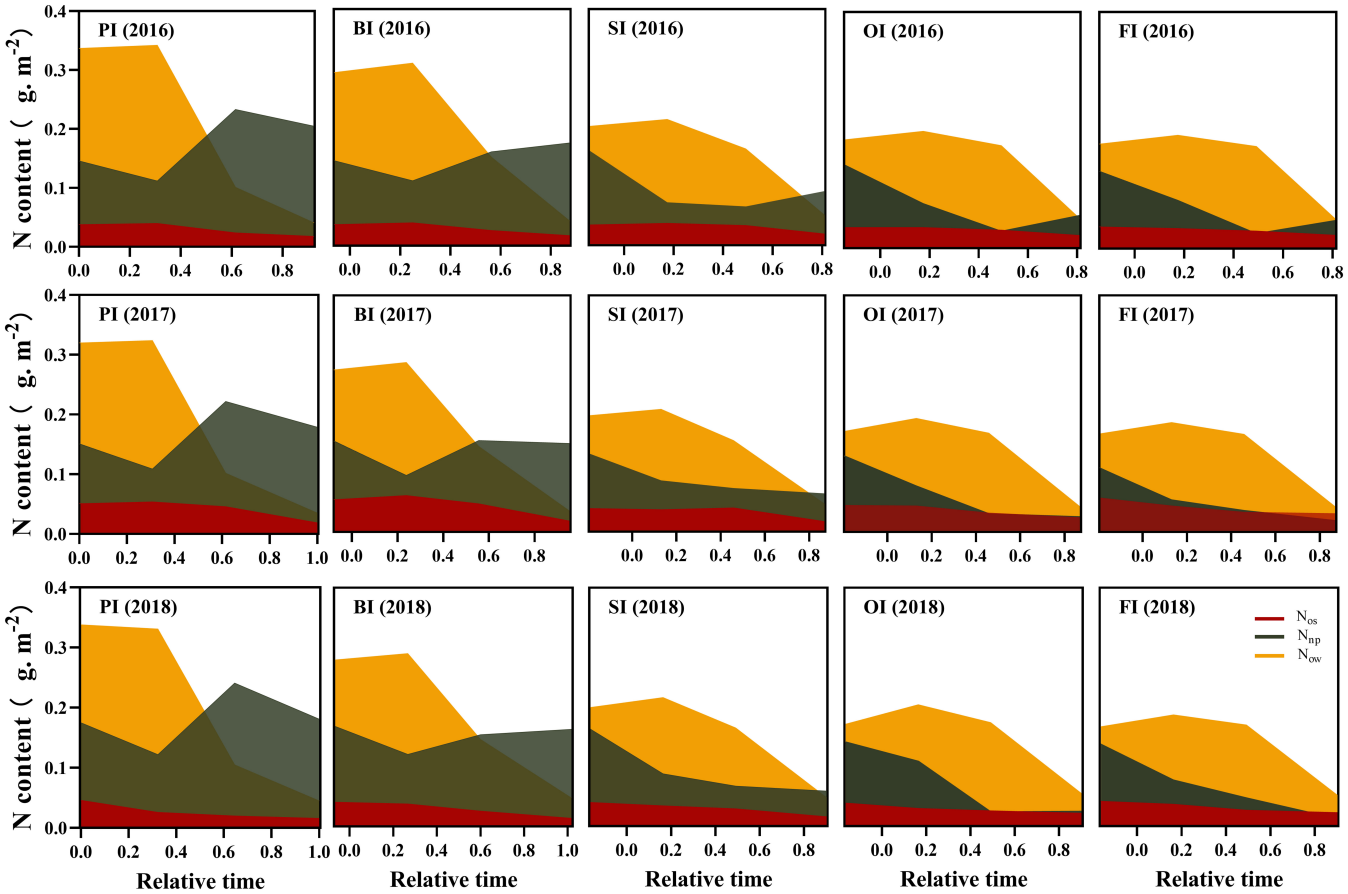
Leaf storage N dynamics during the reproductive period. PI and BI represent drip irrigation under plastic film mulch and biodegradable film mulch, respectively; SI, drip irrigation incorporating straw returning; OI, drip irrigation with the tape buried to a shallow soil depth; FI, furrow irrigation. Relative time is the ratio of days after silking to the duration from silking to maturity. *N*_os_, SDS-soluble protein storage N. *N*_ow_, water-soluble protein storage N. *N*_nop_, non-protein storage N. The data in this figure are presented in **Table S4** for further interpretation.

**Figure 6:**
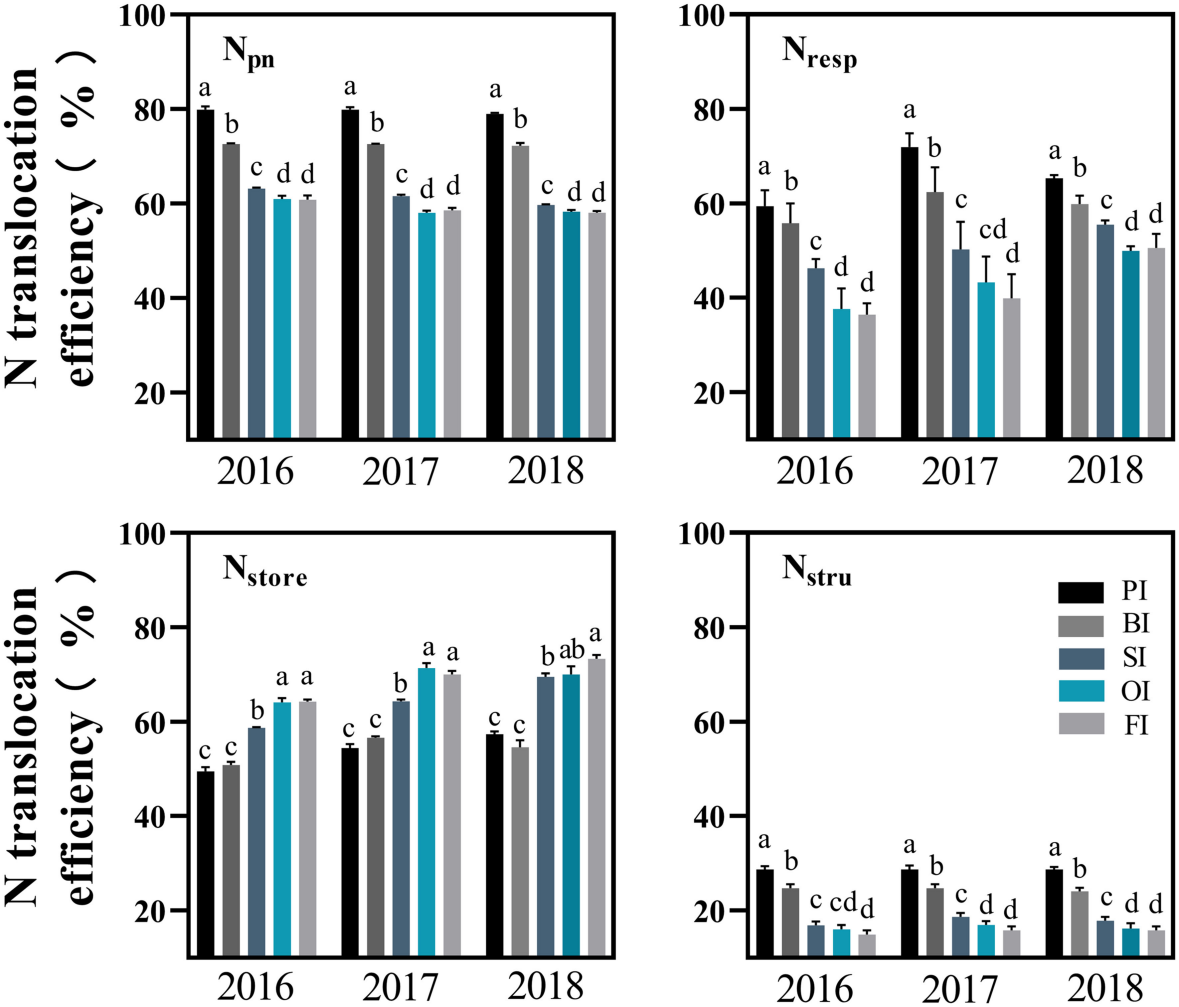
Translocation efficiency of different leaf N components. PI and BI represent drip irrigation under plastic film mulch and biodegradable film mulch, respectively; SI, drip irrigation incorporating straw returning; OI, drip irrigation with the tape buried to a shallow soil depth; FI, furrow irrigation. *N*_pn_, photosynthetic protein N. *N*_resp_, respiration protein N. *N*_stru_, structural protein N. *N*_store_, storage N. The error bars represent standard deviations. Different lowercase letters indicate significant differences at *P* < 0.05.

At earlier filling stage, *N*_pn_ (*N*_Rubisco_, *N*_PEPC_, *N*_PPDK_, *N*_et_, and *N*_lc_), *N*_stru_, *N*_resp_, *N*_ow_, and *N*_os_ ranked as follows: PI > BI > SI > OI, FI (**Figures 3**–**5**). With the filling progress, the improvements for *N*_pn_, *N*_stru_, *N*_resp_, *N*_ow_, and *N*_os_ with PI and BI were weakened due to the protein degradation and higher translocation efficiencies of *N*_pn_, *N*_stru_, and *N*_resp_ (**Figure 6**). Instead, there was an increasement in *N*_nop_ under PI and BI during the late reproductive stage, and SI achieved higher *N*_pn_, *N*_stru_, *N*_resp_, *N*_ow_, and *N*_os_ during the late reproductive stage. In contrast to film mulching and straw returning treatments, OI and FI significantly increased the translocation efficiencies of *N*_store_ (*P<* 0.05).

### Leaf senescence

**Figure 7:**
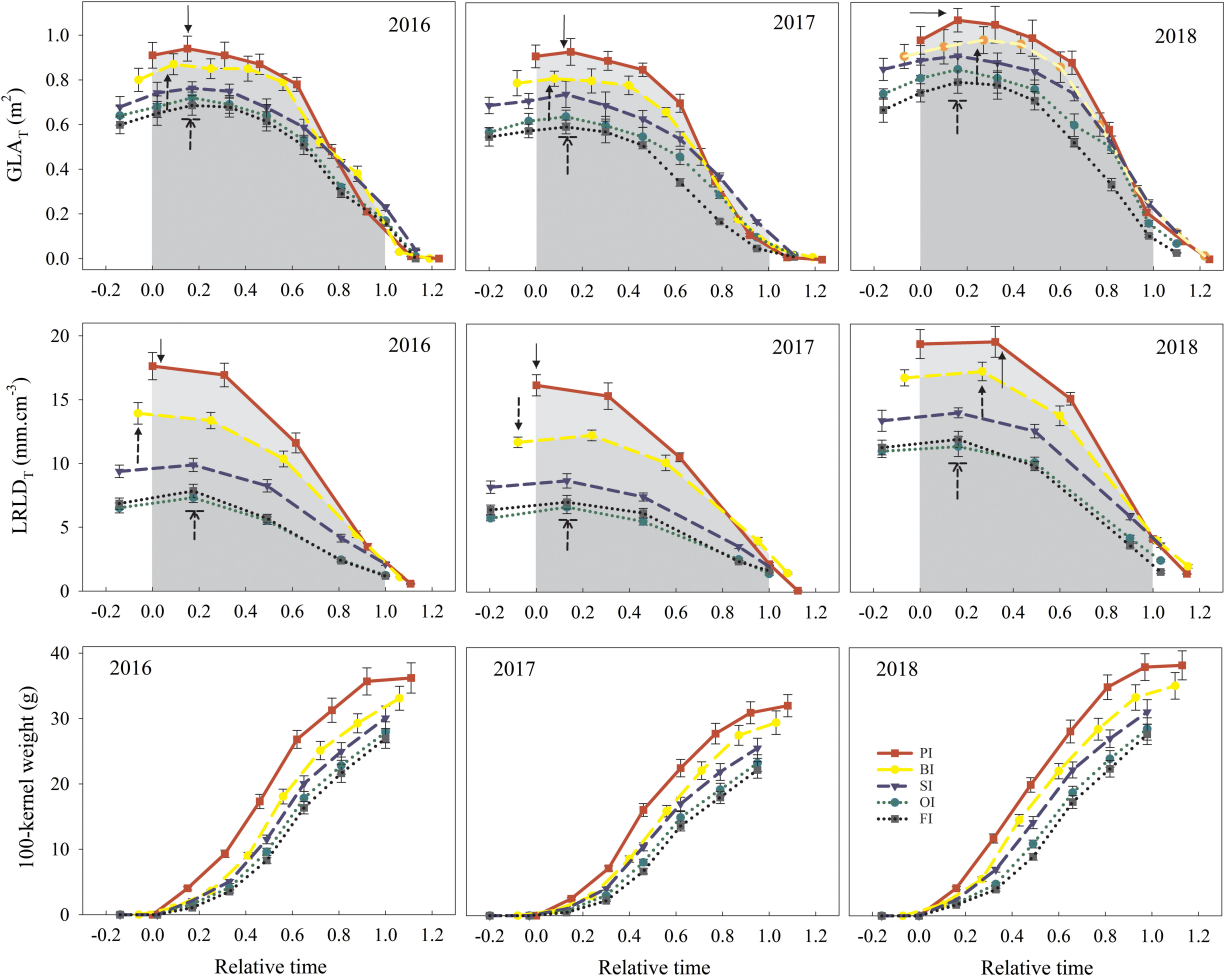
Total green leaf area per plant (*GLA*_T_), total live root length density per plant (*LRLD*_T_), and 100-kernel weight dynamics during the reproductive period. PI and BI represent drip irrigation under plastic film mulch and biodegradable film mulch, respectively; SI, drip irrigation incorporating straw returning; OI, drip irrigation with the tape buried to a shallow soil depth; FI, furrow irrigation. Relative time is the ratio of days after silking to the duration from silking to maturity. Gray area indicates the integral value of GLAT and LRLDT from silking (relative time = 0) to maturity (relative time = 1). Solid arrow for PI, dotted arrow for BI, and dotted arrow for SI; OI and FI with a line indicate the GLAmax or LRLDmax. The error bars represent standard deviations.

PI followed by BI achieved the highest *I_leaf_* associated to the large *GLA*_max_, and the *GLA_T_* value at the beginning of reproductive stage (**Figure 7**). Then, the *GLA_T_* under PI and BI was gradually decreased and even lower than the other treatments considering the fast rate of leaf senescence (*LV*_max_ and *LV*_a_) (**Table 3**). PI delayed the onset time of leaf senescence (*LT*_o_) and simultaneously advanced the terminal time of leaf senescence (*LT*_e_) and then resulted in a short GLA duration (*D_leaf_*). SI averagely prolonged *D_leaf_* by 12, 8, 4, and 7 days compared with PI, BI, OI, and FI, respectively, which maintained a higher *GLA_T_* value at the late of reproductive stage.

**Table 3:** Leaf senescence traits with the different drip irrigation treatments.

Year	Treatments ^a^	*LT_o_* ^b^ (DS)	*LT_e_* (DS)	*D_leaf_* (d)	*LV_max_* (m^2^. d^-1^)	*LV_a_* (m^2^. d^-1^)	*I_leaf_* (m^2^. d)
2016	PI	30.97 a ^c^	81.65 d	50.68 d	0.034 a	0.017 a	46.12 a
	BI	30.38 a	85.18 c	54.81 c	0.029 b	0.015 b	42.98 b
	SI	28.74 b	93.09 a	64.35 a	0.022 c	0.011 c	38.32 c
	OI	27.56 c	87.57 c	60.01 b	0.022 c	0.011 c	33.94 d
	FI	27.62 c	85.86 c	58.24 b	0.021 c	0.011 c	32.26 d
2017	PI	28.96 a	75.67 c	46.71 c	0.037 a	0.018 a	42.66 a
	BI	26.04 b	78.29 b	52.25 b	0.029 b	0.014 b	36.91 b
	SI	23.97 b	85.91 a	61.93 a	0.022 c	0.011 c	33.05 c
	OI	25.45 b	80.16 b	54.71 b	0.021 c	0.010 c	27.81 d
	FI	20.96 c	73.40 d	52.44 b	0.021 c	0.010 c	23.75 e
2018	PI	32.54 a	81.19 cd	48.66 c	0.040 a	0.020 a	52.06 a
	BI	29.58 b	82.93 c	53.35 b	0.034 b	0.017 b	46.88 b
	SI	30.33 b	88.06 a	57.73 a	0.029 c	0.014 c	44.82 bc
	OI	30.18 b	85.06 b	54.88 ab	0.027 c	0.014 c	39.80 cd
	FI	27.04 c	79.47 d	52.44 b	0.027 c	0.013 c	34.93 d

a PI and BI represent drip irrigation under plastic film mulch and biodegradable film mulch, respectively; SI, drip irrigation incorporating straw returning; OI, drip irrigation with the tape buried at a shallow soil depth; FI, furrow irrigation.

b LT_o_, onset of leaf senescence; LT_e_, terminal of leaf senescence; D_leaf_, green leaf duration; LV_max_, maximum leaf senescence rate; LV_a_, average leaf senescence rate; I_leaf_, green leaf integral; DS, days after silking.

c Values are estimated from the Equation 3 fitted to the total green leaf area per plant, and the determination coefficient (R^2^) of the regression equations with different treatments were >0.978. Different lowercase letters indicate significant differences at P < 0.05.

### Root senescence

PI followed by BI maintained a higher *LRLD*_max_, *LRLD_T_* before maturity (**Figure 7**), and resulted in an increased *I_root_* compared with other treatments (**Table 4**). Although PI accelerated maize growth and development progress as well as root senescence rate (*RV*_max_ and *RV*_a_), the onset time of root senescence (*RT*_o_) with PI was only advanced in the drought year of 2017, and it was delayed in both 2016 and 2018 compared with other treatments. The shortened *D_root_* under PI was mainly due to the advanced terminal time of root senescence (*RT*_e_). SI maintained the longest *D_root_*, which postponed the root senescence in contrast to PI. OI significantly delayed *RT*_e_ compared with FI, but no significant differences were found in the other root senescence traits between OI and FI (*P >* 0.05).

**Table 4:** Root senescence traits with the different drip irrigation treatments.

Year	Treatments ^a^	*RT_o_* ^b^ (DS)	*RT_e_* (DS)	*D_root_* (d)	*RV_max_* (mm.cm^-3^. d^-1^)	*RV_a_* (mm.cm^-3^. d^-1^)	*I_root_* (mm.cm^-3^. d)
2016	PI	18.86 a ^c^	88.30 d	69.43 b	0.484 a	0.242 a	801.01 a
	BI	17.66 a	93.21 b	75.55 a	0.348 b	0.174 b	635.78 b
	SI	17.87 a	96.34 a	78.47 a	0.235 c	0.117 c	452.42 c
	OI	15.11 b	92.40 b	77.30 a	0.171 d	0.085 d	306.28 d
	FI	14.45 b	90.09 c	75.64 a	0.185 d	0.092 d	317.46 d
2017	PI	18.73 b	87.75 c	69.02 b	0.445 a	0.222 a	728.51 a
	BI	24.65 a	95.05 a	70.41 a	0.324 b	0.161 b	598.09 b
	SI	19.72 b	95.58 a	75.86 a	0.211 c	0.105 c	396.50 c
	OI	21.68 ab	92.97 ab	71.29 a	0.164 d	0.082 d	292.37 d
	FI	20.91 ab	91.55 b	70.64 a	0.182 d	0.091 cd	315.47 d
2018	PI	25.43 a	89.32 c	63.90 b	0.583 a	0.291 a	957.32 a
	BI	21.73 ab	93.19 b	71.45 a	0.456 b	0.227 b	811.34 b
	SI	23.64 ab	97.42 a	73.78 a	0.356 c	0.178 c	679.78 c
	OI	22.78 ab	93.65 b	70.88 a	0.301 d	0.150 d	539.93 d
	FI	19.43 b	89.72 c	70.29 a	0.314 d	0.157 d	528.92 d

a PI and BI represent drip irrigation under plastic film mulch and biodegradable film mulch, respectively; SI, drip irrigation incorporating straw returning; OI, drip irrigation with the tape buried at a shallow soil depth; FI, furrow irrigation.

b RT_o_, onset of root senescence; RT_e_, terminal of root senescence; D_root_, live root duration; RV_max_, maximum root senescence rate; RV_a_, average root senescence rate; I_root_, live root integral; DS, days after silking.

c Values are estimated from the Equation 3 fitted to the total live root length density per plant, and the determination coefficient (R^2^) of the regression equations with different treatments were >0.975. Different lowercase letters indicate significant differences at P < 0.05.

### Grain filling characteristics

The grain weight during the reproductive stage ranked as follows: PI > BI > SI > OI > FI (**Figure 7**). PI obtained the highest *GW*_max_ and grain filling rate (*GV*_max_ and *GV*_a_). Moreover, PI obviously advanced the onset time of active grain filling period (*GT*_o_) and the terminal time of active grain filling period (*GT*_e_) and then shortened the active grain filling duration (*D_filling_*) compared with other treatments. SI gained higher *GWA* during active grain filling stage compared with OI and FI, attributed to the higher *GV*_max_, *GV*_a_, and *D_filling_* (**Table 5**).

**Table 5:** Grain filling traits with the different drip irrigation treatments.

Year	Treatments ^a^	*GT_o_* ^b^ (DS)	*GT_e_* (DS)	*D_filling_* (d)	*GV_max_* (g. d^-1^)	*GV_a_* (g. d^-1^)	*GWA* (g)
2016	PI	5.04 d ^c^	64.29 c	59.25 b	0.920 a	0.621 a	33.83 a
	BI	10.74 c	71.34 b	60.61 ab	0.841b	0.567 b	31.56 b
	SI	11.15 c	73.74 ab	62.59 a	0.777 c	0.524 c	30.15 c
	OI	12.65 b	74.25 ab	61.60 ab	0.737 c	0.498 c	28.16 d
	FI	14.69 a	77.35 a	62.66 a	0.721 c	0.486 c	27.93 d
2017	PI	8.39 d	68.90 c	60.51 b	0.823 a	0.548 a	30.46 a
	BI	10.14 c	73.08 b	62.93 ab	0.755 b	0.507 b	29.31 b
	SI	10.69 c	76.25 a	65.56 a	0.681 c	0.449 c	27.02 c
	OI	12.47 b	77.09 a	64.61 a	0.632 c	0.419 c	24.88 d
	FI	14.70 d	76.89 a	62.19 ab	0.631 c	0.419 c	23.93 d
2018	PI	5.36 d	68.42 c	63.06 b	0.953 a	0.636 a	36.86 a
	BI	7.68 c	73.00 b	65.31 ab	0.870 b	0.575 b	34.49 b
	SI	8.70 c	76.09 a	67.39 a	0.782 c	0.520 c	32.22 c
	OI	10.72 b	77.19 a	66.48 a	0.734 c	0.493 c	30.15 d
	FI	12.12 a	77.65 a	65.53 ab	0.730 c	0.493 c	29.65 d

a PI and BI represent drip irrigation under plastic film mulch and biodegradable film mulch, respectively; SI, drip irrigation incorporating straw returning; OI, drip irrigation with the tape buried at a shallow soil depth; FI, furrow irrigation.

b GT_o_, onset of active grain filling period; GT_e_, terminal of active grain filling period; D_filling_, active grain filling duration; GV_max_, maximum grain filling rate; GV_a_, average grain filling rate; GWA, grain weight increment during active filling period; DS, days after silking.

c Values are estimated from the Equation 4 fitted to the 100-kernel weight, and the determination coefficient (R^2^) of the regression equations with different treatments were >0.998. Different lowercase letters indicate significant differences at P < 0. 05.

### Senescence parameters related to yield, WUE, and NUE

**Figure 8:**
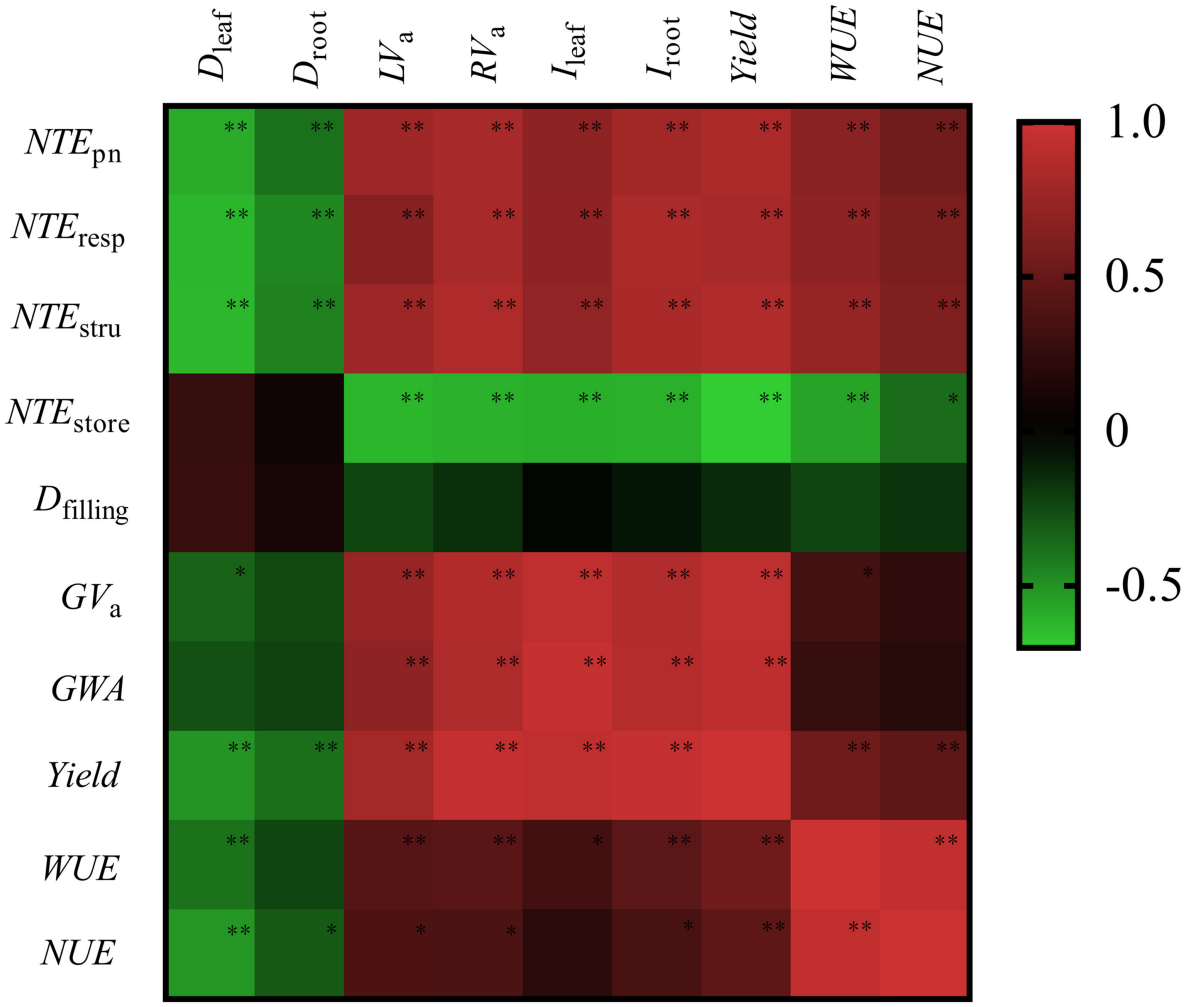
Heatmap of correlation coefficients between senescence traits, filling traits, yield, water, and N use efficiency. *D_leaf_*, green leaf duration; *D_root_*, live root duration; *LV_a_*, average leaf senescence rate; *RV_a_*, average root senescence rate; *I_leaf_*, green leaf integral; *I_root_*, live root integral; *D_filling_*, grain filling duration; *GV_a_*, average grain filling rate; *GWA*, grain weight growth during filling. WUE, water use efficiency. NUE, N use efficiency. NUE_pn_, *NTE*_resp_, *NTE*_stru_, and *NTE*_store_ are the translocation efficiency of photosynthetic N, respiration N, structure N and storage N, respectively. * *P* < 0.05 and ** *P* < 0.01.

High *GWA, GV_a_*, yield, WUE, and NUE were highly positively related to fast senescence rate of source organs (*RV*_a_ and *LV*_a_), large *I_leaf_* and *I_root_*, and high translocation efficiency of leaf protein N (*N*_pn_, *N*_stru_, and *N*_resp_) (*P<* 0.05) (**Figure 8**). *D_root_* and *D_leaf_* did not play a significant role in determining *D_filling_* significantly (*P* > 0.05), which negatively related to yield, WUE, and NUE (*P<* 0.05). The high translocation efficiency of *N*_store_ was not positively associated with yield, WUE, and NUE.

## Discussion

### Drip irrigation combined with film mulching improved *GLA*, *LRLD*, and yield

Previous studies have been proved that film mulching combined with irrigation can obviously promote maize leaf area, root size, and biomass accumulation (Qin et al., 2016; Xue et al., 2017; Wang et al., 2021). Whereas, activated source organs, e.g., GLA and LRLD, during the reproductive stage essentially determine C assimilation, soil water, inorganic nutrient absorption, and thus ultimate grain yield formation. Drip irrigation combined with film mulching achieved the highest *GLA*_T_ and *LRLD*_T_ at the silking stage due to better soil water and temperature conditions (Bu et al., 2013; Wu et al., 2021). In present study, roots were mainly distributed in the 0- to 20-cm soil layers considering the heavy clay soil (Wang et al., 2009; Sampathkumar et al., 2012; Sharma et al., 2017). PI significantly improved LRLD in the 0- to 20-cm soil layers due to the better soil hydrothermal environment during the vegetative stage (**Supplementary Figures 3, 4**). In addition, it facilitates plant growth and contributed to higher *GLA*_T_ and *LRLD*_T_ values compared with other treatments. However, PI decreased LRLD in the deeper 20- to 100-cm soil layers accompanied with lower soil water content, in contrast to BI. SI was particularly beneficial to increase LRLD in the 60- to 100-cm soil layers, which can be explained that straw returning to the field was beneficial to improve soil water and soil structure in the deep soil layers (Wu et al., 2021). FI got higher LRLD than OI in the 20- to 60-cm soil layers, due to more irrigation water percolated to the deeper soil layers (Hassanli et al., 2009). PI and BI showed relatively low *GLA_T_* and *LRLD_T_* only during the late filling stage (around R_5_ stage) compared with other treatments and then maintained high *I_leaf_* and *I_root_* values during the entire reproductive stage.

Yang et al. (2016) found that drip irrigation with plastic film mulching accelerated plant senescence owing to the decreased N supply to canopy, considering the constrictive root architecture and higher soil temperature during the reproductive stage. While some studies pointed out that large root was not required for high yielding potential in high input cropping systems (Sharma et al., 2017). The present study also found that drip irrigation combined with film mulching system, especially PI, improved leaf and root senescence rates with a more constrictive LRLD distribution and higher soil temperature during the reproductive stage compared to other treatments (**Supplementary Figure 4**). We agreed with Christopher et al. (2016) that the higher *I_leaf_* and *I_root_* under drip irrigation combined with film mulching system led to a greater grain yield. *I_leaf_* and *I_root_* had the highest correlation to maize yield in different water environments, which were the most useful indicator to describe plant stay-green trait.

### Drip irrigation combined with film mulching accelerated plant senescence accompanied with large amount of leaf protein N translocation

Maize root stops growing at anthesis when senescence started. We further found that root senescence precedes leaf senescence by 7-10 days averagely with different treatments, which was similar with the result obtained by Lisanti et al. (2013). Root activity depends on C supply from the leaves. While leaf senescence could also be induced by the ageing root, in terms of deficit N/water supply, cytokinin signal molecule, and decreased root respiration (Glanz-Idan et al., 2020; Liu et al., 2018b; Tang et al., 2019). However, in contrast to nature programmed cell death, nucleus and mitochondria remain active for a long time during the senescence process (Roberts et al., 2012), the communications between photosynthesizing leaves and roots still need more investigation during the crop reproductive stage. Drought or low N input could accelerate the senescence process (Pommel et al., 2006; Wang et al., 2013; Christopher et al., 2016). In present study, plant senescence was not induced by drought or low N stresses considering the supplemental irrigation and sufficient fertilizer supply in each treatment. The time of anthesis is a highly variable character and can strongly confound the effect of senescence on productivity (Bogard et al., 2011; Naruoka et al., 2012). PI advanced maize anthesis, but vigorous plant growth at the early filling stage delayed leaf and root senescence onset. In addition, only the advanced root senescence onset time under PI was observed during the year of 2017. Fast senescence under PI followed by BI can be also triggered by the large grain sink and greater nutrient requirement that enhanced N mobilization from source organs to grains (Aubry et al., 2008; Distelfeld et al., 2014; Ma and Dwyer, 1998; Rajcan and Tollenaar, 1999). As a consequence, the leaf protein N contents fell rapidly after silking. The delayed onset time as well as the advanced terminal time of senescence resulted in a short duration of GLA and LRLD under PI and BI. No significant differences were found between OI and FI in senescence rates, GLA and LRLD durations, or filling dynamics due to the approximate soil environment and plant growth process.

Many genetic studies have suggested that the stay-green trait (referred to as a delay in the onset of leaf senescence, or a longer green area duration) correlates with high yield for cereal crops. The previous results also suggested that stay-green cultivars enhanced root absorption capacity for soil water and N nutrient by ensuring the supply of photosynthetic C assimilate (Ma and Dwyer, 1998; Hoang and Kobata, 2009; Bogard et al., 2011; Gaju et al., 2011; Gregersen et al., 2013; Zhang et al., 2013; Liu et al., 2021). However, the relationship between senescence and crop productivity is complex. More recent works showed that there was no consistent advantage of the delayed senescence hybrids on crop production, and stay-green trait could be only necessary for higher yield under terminal drought or low N stresses (Borrell et al., 2000; Acciaresi et al., 2014; Antonietta et al., 2014; Christopher et al., 2016). Moreover, the average temperature at the late filling stage (September) was only 16.8°C, which limited photosynthetic C and N assimilation and slowed the export of nutrients to grains (temperatures between 22-24°C are optimal for maize filling) (Christopher et al., 2016). Therefore, longer GLA and LRLD durations under SI treatment did not contribute to higher yield. We agreed with Xie et al. (2016); Yang and Udvardi (2018) and Zhang et al. (2019) that faster senescence led to better utilization of photosynthetic C and N assimilation for larger grains. Thus, filling rate, grain weight increment, yield, WUE, and NUE were positively associated with senescence rates of leaf and root, but negatively associated with GLA and LRLD durations. We also found that a larger biomass translocation amount and a higher biomass translocation efficiency (**Supplementary Figure 5**) were necessary for high-yield formation under PI and BI during the fast senescence process. In addition to leaf protein N, PI showed a higher biomass translocation amount/efficiency in contrast to BI, which lead to a higher grain weight.

### Higher leaf storage N transport efficiency did not attribute to high yield, WUE, and NUE

**Figure 9:**
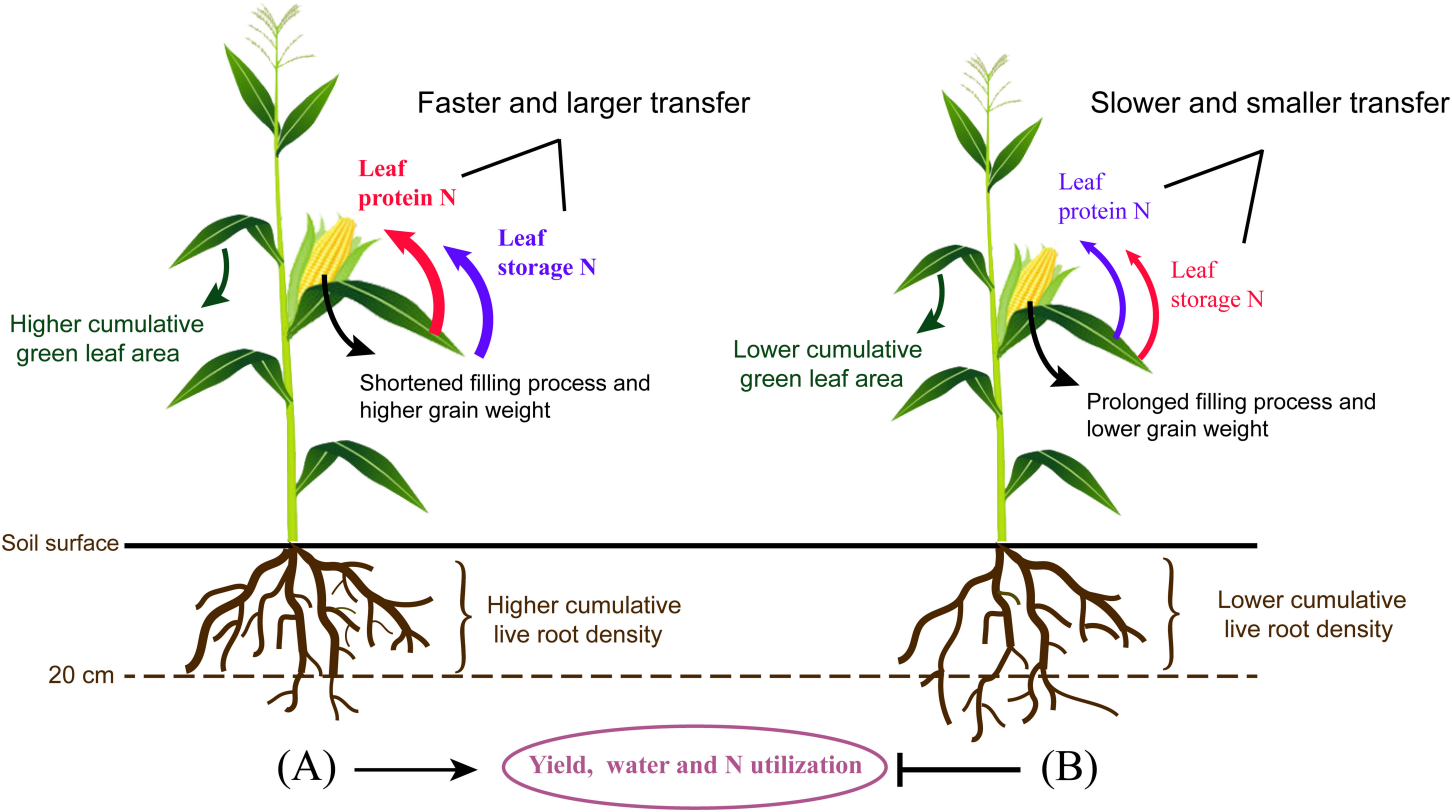
The comparison of drip irrigation under film mulch **(A)** and drip irrigation incorporating straw returning **(B)** during the reproductive stage of maize. In contrast to **(B)**, **(A)** showed a higher cumulative green leaf area and live root density in the top 0- to 20-cm soil layers, but a lower cumulative live root density in the deeper soil layers. In addition, larger N was fast transferred from leaves to grains, accompanied with a shortened filling process and higher grain weight, which contributed to the improvement of yield, water, and N utilization for **(A)**. Non-protein storage N transfer was stimulated by **(B)** to make up for the relative inadequacy of leaf N. In the comparison between **(A, B)**, the red and bule font represent higher and lower translocation efficiency for different N components, respectively.

CO_2_ assimilation capacity is positively regulated by leaf N, which is the main component of chlorophyll and photosynthetic proteins. The distribution of different leaf N fractions determines leaf growth and photosynthesis capacity, thus affecting N utilization. A decrease in photosynthetic rate is mainly due to the degradation of photosynthetic enzymes. Our results showed that different leaf N components decreased along with a reduction of GLA during the reproductive stage. Considering the vigorous vegetative growth, higher *N*_Rubisco_, *N*_PEPC_, *N*_PPDK_, *N*_et_, *N*_lc_, *N*_stru_, *N*_resp_, *N*_ow_, and *N*_os_ were obtained by PI and BI at the earlier filling stage. The transfer of leaf N after anthesis has an important effect on photosynthesis. In addition, degraded leaf proteins provided an enormous source of N for kernel development (Masclaux-Daubresse et al., 2010). Mu et al. (2018) found that photosynthetic proteins, i.e., Rubisco, PEPC, and PPDK, had great transfer potential in maize, and their transfer efficiencies were enhanced by low N treatment. Storage N in the forms of nitrate, amino acid, and protein is important for plant to prevent from adversity (Xu et al., 2012; Liu et al., 2018a). However, the regulation effect of storage N on crop production during the senescence period is still lack of research. Our results further showed that the highest translocation efficiency was found in *N*_pn_, whereas the lowest was found in *N*_stru_. In contrast to PI, SI improved soil water and achieved higher LRLD in the deep soil layer, which was beneficial to root absorption capacity, therefore allowing leaves to retain photosynthetic capacity with less N mobilization during the reproductive period, and led to a higher leaf N content during the late reproductive stage. PI followed by BI had the highest translocation efficiency in *N*_pn_, *N*_resp_, and *N*_stru_, which were positively associated with senescence rate (*LV*_a_ and *RV*_a_), NUE, WUE, and yield and were negatively correlated with *D*_leaf_ and *D*_root_. Meanwhile, non-protein storage N accumulated only under PI and BI treatments during the late reproductive stage. PI and BI showed a low translocation efficiency of *N*_store_ compared with SI, OI, and FI. Thus, it can be concluded that higher remobilization of non-protein storage N was improved by SI, FI, and OI to make up for the relative inadequacy of leaf N. Faster and larger translocation of protein N from leaves to grains ensured a high WUE and NUE under drip irrigation combined with film mulching system (**Figure 9**).

## Conclusions

Drip irrigation combined with a film mulching system achieved the highest grain yield, WUE, and NUE, by increasing the cumulative GLA and LRLD, biomass, and leaf protein N transportation efficiency during the reproductive period. Drip irrigation combined with biodegradable film mulching had no significant differences in yield, WUE, and NUE compared to that with plastic film mulching, and it is the recommended practice to reduce overall use of plastic and creation of plastic waste. Under drip irrigation combined with returning straw into soil, root growth was effectively promoted in the deeper soil layer, and the duration of GLA and LRLD was prolonged. However, the delayed senescence under this system did not contribute to higher yield, considering the limited C and N assimilation capacity associated with low air temperature during the late reproductive stage in the northeast plain of China. Larger remobilization of leaf non-protein storage N could not contribute to a high yield, WUE, or NUE under drip irrigation combined with straw returning, drip irrigation with the tape buried at a shallow soil depth, and furrow irrigation systems. Whereas, the hormone signals and molecular regulation mechanisms of the protein N translocation from leaves to grains are worthy of further exploration in the future under different cropping systems.

## Data availability statement

The raw data supporting the conclusions of this article will be made available by the authors, without undue reservation.

## Author contributions

YW and YJW designed the study. YW, FYY, and XNL performed the measurements. LM performed the data analysis and wrote the first draft of the manuscript, which was extensively edited by all authors. All authors contributed to the article and approved the submitted version.
