# Supplementary material for: Association of maize (Zea mays L.) senescence with water and nitrogen utilization under different drip irrigation systems
Source: Front Plant Sci. 2023 Mar 17;14:1133206. doi: 10.3389/fpls.2023.1133206 (PMC10064151; doi:10.3389/fpls.2023.1133206)
Supplement: Supplementary file 1 [file DataSheet_1.docx]

Supplementary Material


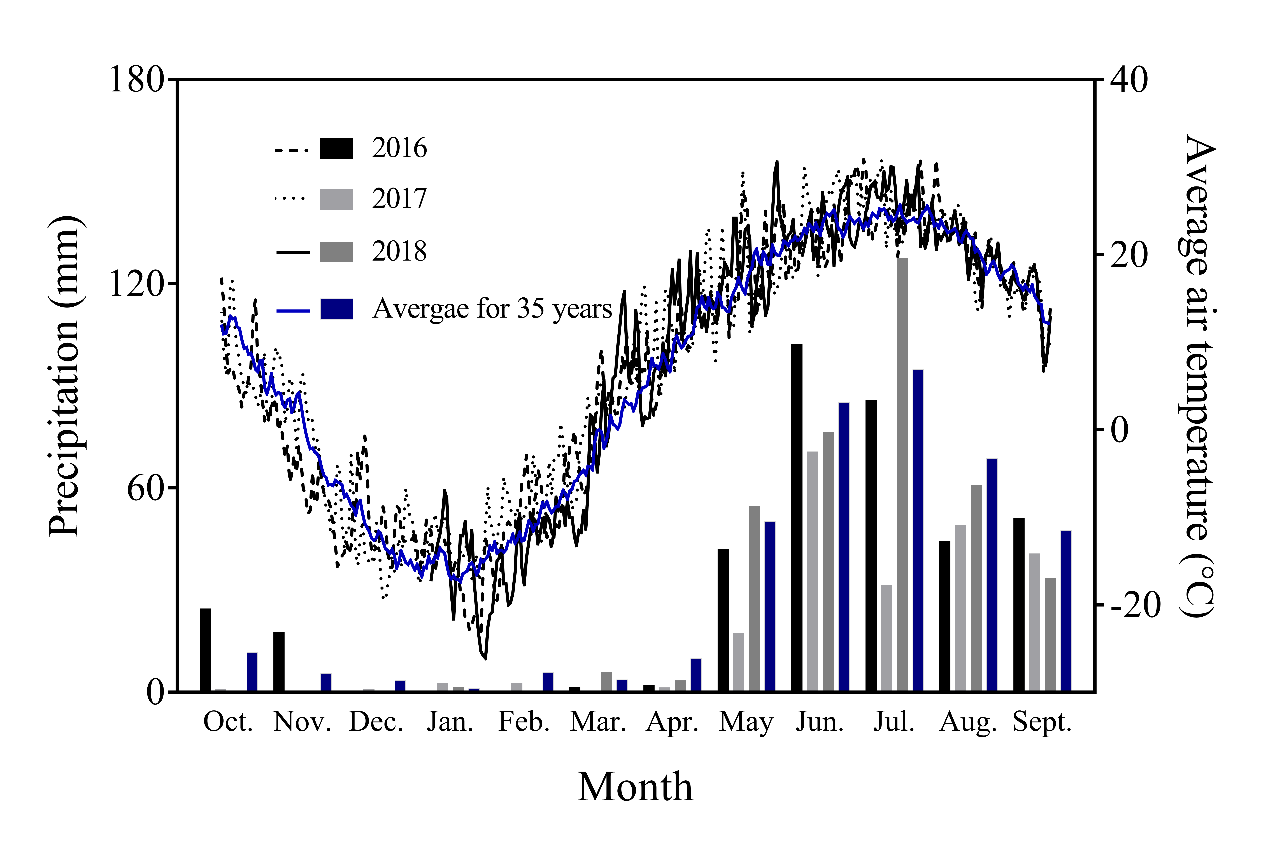


**Supplementary Figure 1.** Total monthly precipitation and daily air temperature distribution for the 2016-2018 growing seasons at the experimental site.





**Supplementary Figure 2.** Schematic diagram of the ﬁeld layout. Each grey square of dotted lines represents the sampled area of the root system. Red dots are the soil water sample positions.



**Supplementary Figure 3.** Soil water content in the 0-100 cm soil layer at jointing (A), silking (B), filling (C) and maturity (D) stage.

Note: PI and BI represent drip irrigation under plastic film mulch and biodegradable film mulch, respectively; SI, drip irrigation incorporating straw returning; OI, drip irrigation with the tape buried to a shallow soil depth; FI, furrow irrigation. The error bars represent standard deviations.

**
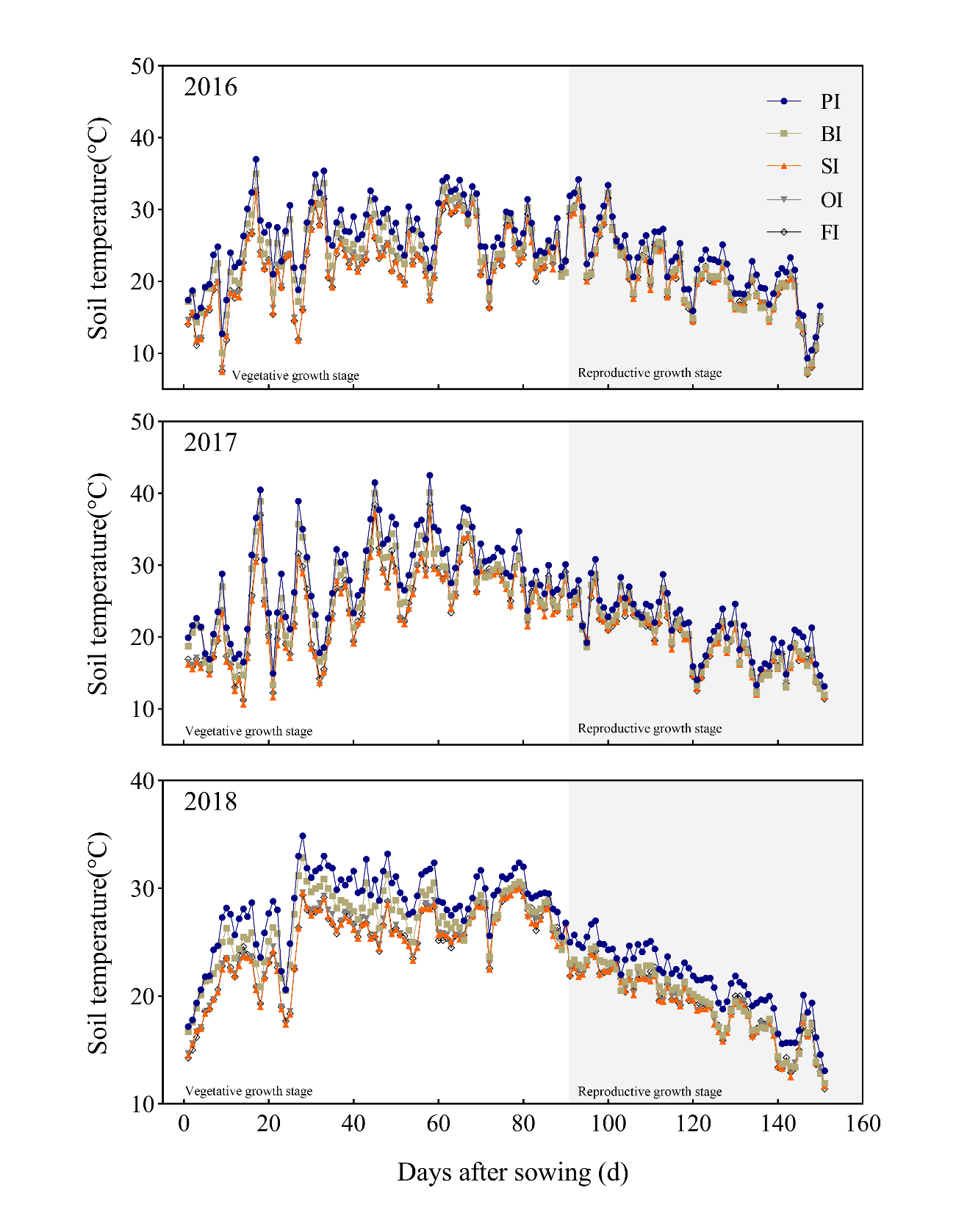
Supplementary Figure 4.** Soil temperature in the 0-10 cm soil layer during the growth period.

Note: PI and BI represent drip irrigation under plastic film mulch and biodegradable film mulch, respectively; SI, drip irrigation incorporating straw returning; OI, drip irrigation with the tape buried to a shallow soil depth; FI, furrow irrigation. The error bars represent standard deviations.


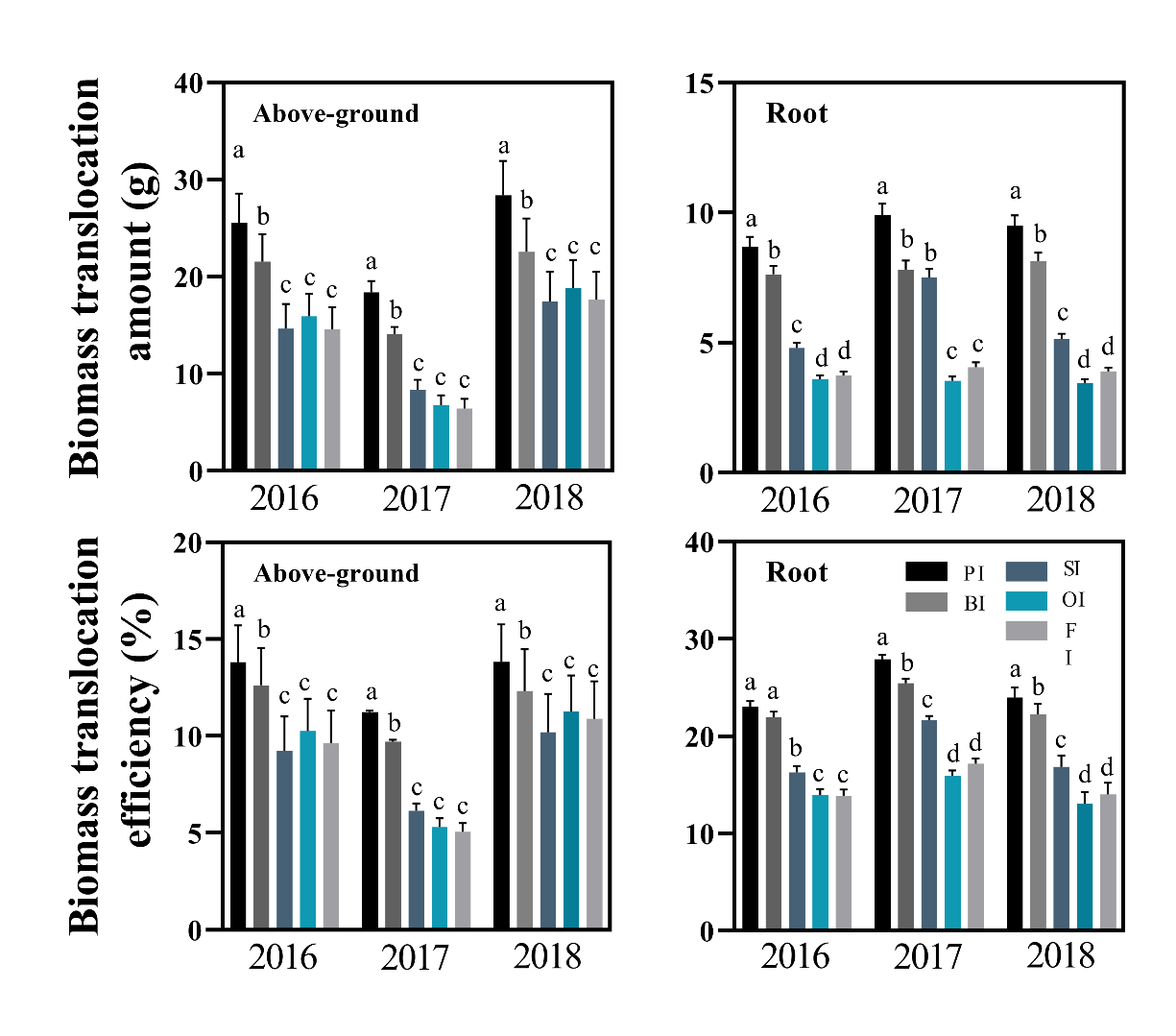


**Supplementary Figure 5.** Translocation amount and efficiency of maize plant biomass.

Note: PI and BI represent drip irrigation under plastic film mulch and biodegradable film mulch, respectively; SI, drip irrigation incorporating straw returning; OI, drip irrigation with the tape buried to a shallow soil depth; FI, furrow irrigation. The error bars represent standard deviations. Different lowercase letters indicate significant differences at *P* < 0.05.

**Supplementary Table 1. Monthly precipitation and air temperature during the experimental period.**

| Rainfall (mm) | May | June | July | August | September |
| --- | --- | --- | --- | --- | --- |
| 2016 | 42.2 | 102.2 | 85.5 | 44.5 | 27.4 |
| 2017 | 17.3 | 70.7 | 31.4 | 49.4 | 40.7 |
| 2018 | 54.6 | 76.5 | 127.6 | 60.9 | 25.1 |
| Air temperature (℃) | May | June | July | August | September |
| 2016 | 17.5 | 21.6 | 25.5 | 23.6 | 16.6 |
| 2017 | 17.6 | 23.0 | 26.1 | 22.5 | 16.1 |
| 2018 | 17.2 | 23.2 | 26.0 | 22.4 | 16.0 |

**Supplementary Table 2. Irrigation management during the experimental period.**

| **Year** | **Irrigation and Precipitation events** |  |  |  |  |
| --- | --- | --- | --- | --- | --- |
| 2016 | Irrigation time | 5/2 | 6/7 | 7/5 | 8/16 |
|  | Irrigation amount (mm) | 55 | 40 | 30 | 20 |
|  | Precipitation amount between irrigation events (mm) | - | 43.8 | 100.6 | 91.7 |
| 2017 | Irrigation time | 4/30 | 6/5 | 7/2 | 8/20 |
|  | Irrigation amount (mm) | 55 | 40 | 30 | 20 |
|  | Precipitation amount between irrigation events (mm) | - | 17.3 | 73.0 | 77.6 |
| 2018 | Irrigation time | 5/4 | 6/8 | 7/4 | 8/19 |
|  | Irrigation amount (mm) | 55 | 40 | 30 | 20 |
|  | Precipitation amount between irrigation events (mm) | - | 54.6 | 88.9 | 115.2 |

**Supplementary Table 3. Grain yield, soil water use efficiency, and nitrogen use efficiency for the different irrigation treatments**

| Year | Treatments ^a^ | Yield  (t hm^−2^) | WUE ^b^  (kg mm^–3^) | NUE  (g.g^-1^) | NTE-A  (%) | NTE-R  (%) |
| --- | --- | --- | --- | --- | --- | --- |
| 2016 | PI | 12.65 a ^c^ | 3.17 a | 31.93 a | 48.29 a | 45.97 a |
|  | BI | 11.86 a | 3.10 a | 31.04 ab | 46.72 ab | 44.92 ab |
|  | SI | 10.27 b | 2.84 b | 28.44 bc | 42.83 bc | 43.30 ab |
|  | OI | 9.49 bc | 2.55 c | 28.58 bc | 41.36 c | 42.41 ab |
|  | FI | 9.23 c | 2.46 c | 27.80 c | 39.73 c | 41.12 b |
|  |  |  |  |  |  |  |
| 2017 | PI | 12.42 a | 3.81 a | 37.03 a | 49.56 a | 52.72 a |
|  | BI | 11.51 a | 3.73 a | 36.57 a | 48.73 a | 50.57 ab |
|  | SI | 9.59 b | 3.36 b | 32.05 b | 41.82 b | 49.44 ab |
|  | OI | 8.82 bc | 2.98 c | 32.32 b | 41.24 b | 48.25 ab |
|  | FI | 8.68 c | 2.90 c | 31.59 b | 39.91 b | 46.78 b |
|  |  |  |  |  |  |  |
| 2018 | PI | 13.64 a | 3.15 a | 31.83 a | 46.18 a | 45.91 a |
|  | BI | 12.95 a | 3.09 ab | 31.10 ab | 44.95 ab | 45.04 ab |
|  | SI | 11.19 b | 2.83 bc | 29.63 ab | 41.88 bc | 43.43 ab |
|  | OI | 10.48 bc | 2.58 cd | 29.38 b | 40.53 c | 42.37 ab |
|  | FI | 10.26 c | 2.50 d | 28.95 b | 39.17 c | 42.10 b |

^a^ PI and BI represent drip irrigation under plastic film mulch and biodegradable film mulch, respectively; SI, drip irrigation incorporating straw returning; OI, drip irrigation with the tape buried at a shallow soil depth; FI, furrow irrigation.

^b^ WUE, soil water use efficiency; NUE, nitrogen use efficiency; NTE-A, root nitrogen use efficiency; NTE-R, N translocation efficiency for aboveground plant part; NTE-R, N translocation efficiency for root.

^c^ Different lowercase letters indicate significant differences at *P* < 0. 05.

**Supplementary Table 4. Leaf N** **components (g/m^2^) with different irrigation treatments**

| **Date** | **Treatment** | ***N*_Rubisco_** | ***N*_PEPC_** | ***N*_PPDK_** | ***N*_et_** | ***N*_lc_** | ***N*_resp_** | ***N*_stru_** | ***N*_ow_** | ***N*_os_** | ***N*_nop_** |
| --- | --- | --- | --- | --- | --- | --- | --- | --- | --- | --- | --- |
| 2016/7/17 | PI | 0.160 | 0.047 | 0.029 | 0.202 | 0.319 | 0.012 | 0.133 | 0.337 | 0.038 | 0.146 |
|  | BI | 0.152 | 0.041 | 0.028 | 0.191 | 0.287 | 0.011 | 0.134 | 0.293 | 0.035 | 0.144 |
|  | SI | 0.138 | 0.037 | 0.025 | 0.166 | 0.228 | 0.009 | 0.132 | 0.202 | 0.034 | 0.160 |
|  | OI | 0.125 | 0.029 | 0.022 | 0.161 | 0.200 | 0.007 | 0.127 | 0.184 | 0.035 | 0.141 |
|  | FI | 0.124 | 0.029 | 0.022 | 0.156 | 0.194 | 0.007 | 0.125 | 0.177 | 0.036 | 0.131 |
| 2016/8/6 | PI | 0.153 | 0.042 | 0.029 | 0.172 | 0.283 | 0.011 | 0.136 | 0.342 | 0.040 | 0.112 |
|  | BI | 0.154 | 0.044 | 0.030 | 0.177 | 0.269 | 0.011 | 0.135 | 0.309 | 0.039 | 0.110 |
|  | SI | 0.140 | 0.039 | 0.026 | 0.146 | 0.220 | 0.008 | 0.132 | 0.214 | 0.038 | 0.073 |
|  | OI | 0.126 | 0.031 | 0.022 | 0.141 | 0.190 | 0.007 | 0.129 | 0.198 | 0.035 | 0.076 |
|  | FI | 0.126 | 0.031 | 0.023 | 0.133 | 0.184 | 0.006 | 0.128 | 0.192 | 0.034 | 0.082 |
| 2016/8/26 | PI | 0.078 | 0.021 | 0.015 | 0.107 | 0.183 | 0.009 | 0.106 | 0.101 | 0.023 | 0.233 |
|  | BI | 0.087 | 0.023 | 0.018 | 0.144 | 0.190 | 0.010 | 0.115 | 0.150 | 0.025 | 0.159 |
|  | SI | 0.101 | 0.027 | 0.019 | 0.115 | 0.151 | 0.007 | 0.125 | 0.164 | 0.033 | 0.066 |
|  | OI | 0.083 | 0.021 | 0.016 | 0.102 | 0.126 | 0.006 | 0.121 | 0.174 | 0.032 | 0.029 |
|  | FI | 0.082 | 0.022 | 0.015 | 0.094 | 0.122 | 0.005 | 0.118 | 0.173 | 0.030 | 0.026 |
| 2016/9/15 | PI | 0.041 | 0.011 | 0.008 | 0.044 | 0.049 | 0.005 | 0.095 | 0.041 | 0.017 | 0.205 |
|  | BI | 0.046 | 0.013 | 0.009 | 0.058 | 0.065 | 0.005 | 0.101 | 0.042 | 0.017 | 0.174 |
|  | SI | 0.052 | 0.015 | 0.010 | 0.065 | 0.077 | 0.005 | 0.110 | 0.052 | 0.019 | 0.092 |
|  | OI | 0.052 | 0.013 | 0.010 | 0.065 | 0.070 | 0.004 | 0.107 | 0.051 | 0.022 | 0.056 |
|  | FI | 0.050 | 0.013 | 0.010 | 0.063 | 0.070 | 0.004 | 0.106 | 0.051 | 0.024 | 0.048 |
| 2017/7/13 | PI | 0.150 | 0.044 | 0.028 | 0.188 | 0.300 | 0.008 | 0.124 | 0.320 | 0.036 | 0.151 |
|  | BI | 0.142 | 0.038 | 0.026 | 0.174 | 0.269 | 0.007 | 0.125 | 0.274 | 0.039 | 0.154 |
|  | SI | 0.131 | 0.035 | 0.024 | 0.149 | 0.216 | 0.005 | 0.123 | 0.197 | 0.029 | 0.132 |
|  | OI | 0.119 | 0.027 | 0.021 | 0.144 | 0.186 | 0.004 | 0.118 | 0.171 | 0.038 | 0.129 |
|  | FI | 0.118 | 0.027 | 0.021 | 0.130 | 0.182 | 0.003 | 0.114 | 0.167 | 0.046 | 0.109 |
| 2017/8/2 | PI | 0.144 | 0.040 | 0.027 | 0.149 | 0.264 | 0.007 | 0.126 | 0.324 | 0.041 | 0.109 |
|  | BI | 0.146 | 0.042 | 0.028 | 0.145 | 0.253 | 0.006 | 0.126 | 0.288 | 0.052 | 0.096 |
|  | SI | 0.129 | 0.036 | 0.025 | 0.128 | 0.210 | 0.004 | 0.122 | 0.208 | 0.028 | 0.087 |
|  | OI | 0.119 | 0.029 | 0.021 | 0.115 | 0.180 | 0.003 | 0.117 | 0.193 | 0.036 | 0.078 |
|  | FI | 0.116 | 0.029 | 0.021 | 0.112 | 0.171 | 0.003 | 0.118 | 0.186 | 0.038 | 0.055 |
| 2017/8/22 | PI | 0.072 | 0.020 | 0.014 | 0.077 | 0.172 | 0.005 | 0.099 | 0.102 | 0.039 | 0.222 |
|  | BI | 0.082 | 0.022 | 0.017 | 0.105 | 0.180 | 0.005 | 0.106 | 0.145 | 0.039 | 0.155 |
|  | SI | 0.093 | 0.026 | 0.018 | 0.094 | 0.144 | 0.004 | 0.115 | 0.155 | 0.036 | 0.074 |
|  | OI | 0.077 | 0.020 | 0.015 | 0.084 | 0.117 | 0.003 | 0.110 | 0.168 | 0.028 | 0.031 |
|  | FI | 0.076 | 0.020 | 0.014 | 0.079 | 0.113 | 0.003 | 0.110 | 0.166 | 0.027 | 0.037 |
| 2017/9/11 | PI | 0.039 | 0.011 | 0.007 | 0.039 | 0.046 | 0.002 | 0.088 | 0.035 | 0.016 | 0.179 |
|  | BI | 0.044 | 0.012 | 0.008 | 0.052 | 0.062 | 0.003 | 0.094 | 0.036 | 0.016 | 0.150 |
|  | SI | 0.050 | 0.014 | 0.009 | 0.067 | 0.074 | 0.002 | 0.100 | 0.048 | 0.015 | 0.065 |
|  | OI | 0.049 | 0.012 | 0.009 | 0.074 | 0.064 | 0.002 | 0.098 | 0.044 | 0.027 | 0.026 |
|  | FI | 0.047 | 0.012 | 0.009 | 0.068 | 0.062 | 0.002 | 0.096 | 0.044 | 0.032 | 0.020 |
| 2018/7/18 | PI | 0.156 | 0.044 | 0.029 | 0.216 | 0.328 | 0.016 | 0.127 | 0.338 | 0.058 | 0.175 |
|  | BI | 0.147 | 0.039 | 0.027 | 0.207 | 0.297 | 0.015 | 0.128 | 0.279 | 0.054 | 0.168 |
|  | SI | 0.135 | 0.035 | 0.025 | 0.173 | 0.237 | 0.012 | 0.126 | 0.199 | 0.051 | 0.163 |
|  | OI | 0.125 | 0.027 | 0.021 | 0.170 | 0.206 | 0.011 | 0.123 | 0.171 | 0.050 | 0.140 |
|  | FI | 0.124 | 0.028 | 0.021 | 0.166 | 0.201 | 0.010 | 0.117 | 0.167 | 0.048 | 0.138 |
| 2018/8/7 | PI | 0.150 | 0.040 | 0.028 | 0.190 | 0.294 | 0.014 | 0.133 | 0.331 | 0.042 | 0.122 |
|  | BI | 0.154 | 0.043 | 0.029 | 0.194 | 0.277 | 0.015 | 0.130 | 0.289 | 0.050 | 0.121 |
|  | SI | 0.132 | 0.037 | 0.026 | 0.155 | 0.229 | 0.011 | 0.129 | 0.216 | 0.044 | 0.088 |
|  | OI | 0.125 | 0.030 | 0.022 | 0.146 | 0.194 | 0.010 | 0.122 | 0.204 | 0.039 | 0.108 |
|  | FI | 0.121 | 0.029 | 0.022 | 0.142 | 0.190 | 0.009 | 0.122 | 0.187 | 0.043 | 0.078 |
| 2018/8/27 | PI | 0.074 | 0.020 | 0.014 | 0.117 | 0.190 | 0.011 | 0.104 | 0.105 | 0.029 | 0.241 |
|  | BI | 0.085 | 0.022 | 0.018 | 0.145 | 0.194 | 0.013 | 0.111 | 0.146 | 0.037 | 0.154 |
|  | SI | 0.097 | 0.026 | 0.019 | 0.124 | 0.154 | 0.010 | 0.121 | 0.165 | 0.036 | 0.067 |
|  | OI | 0.081 | 0.021 | 0.015 | 0.115 | 0.129 | 0.009 | 0.114 | 0.174 | 0.031 | 0.023 |
|  | FI | 0.079 | 0.021 | 0.015 | 0.109 | 0.124 | 0.009 | 0.119 | 0.170 | 0.030 | 0.048 |
| 2018/9/16 | PI | 0.041 | 0.011 | 0.008 | 0.052 | 0.050 | 0.005 | 0.091 | 0.045 | 0.018 | 0.181 |
|  | BI | 0.046 | 0.012 | 0.009 | 0.065 | 0.067 | 0.006 | 0.097 | 0.048 | 0.017 | 0.163 |
|  | SI | 0.052 | 0.014 | 0.010 | 0.089 | 0.079 | 0.005 | 0.104 | 0.048 | 0.019 | 0.059 |
|  | OI | 0.050 | 0.013 | 0.009 | 0.090 | 0.067 | 0.005 | 0.103 | 0.055 | 0.029 | 0.024 |
|  | FI | 0.051 | 0.013 | 0.009 | 0.089 | 0.065 | 0.005 | 0.099 | 0.052 | 0.029 | 0.013 |

Note: PI and BI represent drip irrigation under plastic film mulch and biodegradable film mulch, respectively; SI, drip irrigation incorporating straw returning; OI, drip irrigation with the tape buried to a shallow soil depth; FI, furrow irrigation.
